# Effects of exercise in people with multiple sclerosis: a systematic review and meta-analysis

**DOI:** 10.3389/fpubh.2024.1387658

**Published:** 2024-04-10

**Authors:** Liwen Du, Haoyu Xi, Shiyan Zhang, Yilun Zhou, Xifeng Tao, Yuanyuan Lv, Xiao Hou, Laikang Yu

**Affiliations:** ^1^Key Laboratory of Physical Fitness and Exercise, Ministry of Education, Beijing Sport University, Beijing, China; ^2^Department of Strength and Conditioning Assessment and Monitoring, Beijing Sport University, Beijing, China; ^3^School of Physical Education, Xihua University, Chengdu, China; ^4^China Institute of Sport and Health Science, Beijing Sport University, Beijing, China; ^5^School of Sport Sciences, Beijing Sport University, Beijing, China

**Keywords:** exercise, multiple sclerosis, balance, walking ability, walking endurance, fatigue, quality of life

## Abstract

**Background:**

A growing body of studies have examined the effect of exercise in people with multiple sclerosis (MS), while findings of available studies were conflicting. This meta-analysis aimed to explore the effects of exercise on balance, walking ability, walking endurance, fatigue, and quality of life in people with MS.

**Methods:**

We searched PubMed, Web of Science, Scopus, and Cochrane databases, through March 1, 2024. Inclusion criteria were: (1) RCTs; (2) included an intervention and control group; (3) had people with MS as study subjects; (4) had balance, walking ability, walking endurance, fatigue, or quality of life as the outcome measures. Exclusion criteria were: (1) non-English publications; (2) animal model publications; (3) review articles; and (4) conference articles. A meta-analysis was conducted to calculate weighted mean difference (WMD) and 95% confidence interval (CI). Cochrane risk assessment tool and Physiotherapy Evidence Database (PEDro) scale were used to evaluate the methodological quality of the included studies.

**Results:**

Forty studies with a total of 56 exercise groups (*n* = 1,300) and 40 control groups (*n* = 827) were eligible for meta-analysis. Exercise significantly improved BBS (WMD, 3.77; 95% CI, 3.01 to 4.53, *P* < 0.00001), TUG (WMD, −1.33; 95% CI, −1.57 to −1.08, *P* < 0.00001), MSWS-12 (WMD, −2.57; 95% CI, −3.99 to −1.15, *P* = 0.0004), 6MWT (WMD, 25.56; 95% CI, 16.34 to 34.79, *P* < 0.00001), fatigue (WMD, −4.34; 95% CI, −5.83 to −2.84, *P* < 0.00001), and MSQOL-54 in people with MS (WMD, 11.80; 95% CI, 5.70 to 17.90, *P* = 0.0002) in people with MS. Subgroup analyses showed that aerobic exercise, resistance exercise, and multicomponent training were all effective in improving fatigue in people with MS, with resistance exercise being the most effective intervention type. In addition, a younger age was associated with a larger improvement in fatigue. Furthermore, aerobic exercise and multicomponent training were all effective in improving quality of life in people with MS, with aerobic exercise being the most effective intervention type.

**Conclusion:**

Exercise had beneficial effects in improving balance, walking ability, walking endurance, fatigue, and quality of life in people with MS. Resistance exercise and aerobic exercise are the most effective interventions for improving fatigue and quality of life in people with MS, respectively. The effect of exercise on improving fatigue was associated with the age of the participants, with the younger age of the participants, the greater the improvement in fatigue.

**Systematic review registration:**

https://www.crd.york.ac.uk/prospero/display_record.php?RecordID=371056, identifier: CRD42022371056.

## Introduction

Multiple sclerosis (MS) is a disabling neurological disease common in young and middle-aged adults with a mean age of onset of 29 years (1, 2). The manifestations of people with MS include physical symptoms such as muscle weakness, muscle spasms, decreased mobility and balance, and increased sensitivity to pain, with psychiatric episodes and fatigue leading to severe disability and deterioration of physical condition, mobility, cognition, and quality of life (3–7). In fact, 50–80% of people with MS, even in its mild stages, will result in impaired walking performance, further reducing their quality of life as the disease progresses (8).

People with MS usually use pharmacologic strategies that down-regulate immune activation to halt disease progression, prevent relapse, or partially reverse disability (9). However, pharmacologic treatments are often accompanied by adverse effects such as infection, headache, and diarrhea (10). In recent years, exercise has been found to be beneficial in improving aerobic capacity, muscle strength, flexibility, balance, fatigue, and cognitive function in people with MS (11).

A growing body of studies have examined the effect of exercise in people with MS, while findings of available studies were conflicting. Kubsik et al. (12) showed that exercise not only contributes to the physical abilities of people with MS, but also to their mood and attitude toward exercise. In addition, Grazioli et al. (13) reported that multicomponent training was effective in improving quality of life, walking ability, and balance, as well as reducing depression, fatigue, and disease severity in people with MS. Furthermore, Feys et al. (14) showed that running improved aerobic capacity, functional mobility, spatial memory, fatigue, and quality of life in people with MS. However, a meta-analysis showed no significant differences in step count and moderate to vigorous physical activity among individuals with MS, both within and between groups receiving physical activity interventions (14). To the best of our knowledge, Arntzen et al. (15) included only eight randomized controlled trials (RCTs) and the number of included studies was quite small, and the authors included one study in which participants in the control group also received exercise intervention. Another study evaluated the effects of Pilates on balance in people with MS, which included only seven RCTs (16). However, the authors included studies in which control group participants also received exercise interventions such as home exercises (two studies), relaxation exercises (one study), aerobic exercises (one study), and traditional exercises (one study), which may have had some impact on their findings. Therefore, we conducted a comprehensive systematic review and meta-analysis of RCTs to explore the effects of exercise on balance, walking ability, walking endurance, fatigue, and quality of life in people with MS.

## Methods

This systematic review and meta-analysis was done in accordance with the Preferred Reporting Items for Systematic Reviews and Meta-Analysis (PRISMA, 2020) guidelines (17) and the implementing PRISMA in exercise, rehabilitation, sport medicine, and sports science (PERSiST) guidance (18). The protocol was registered with PROSPERO (CRD42022371056).

### Search strategy

We searched the PubMed, Web of Science, Scopus, and Cochrane databases for RCTs relating to the effect of exercise on balance, gait, fatigue, and quality of life in patients with MS from the inception dates to March 1, 2024 (Supplementary Table 1). We also manually searched references listed in the identified systematic reviews and meta-analyses, in addition to the reference lists of identified studies included in the screening. Two authors (L.D. and H.X.) independently completed the article screening using a standardized form.

### Inclusion and exclusion criteria

Inclusion criteria were: (1) RCTs; (2) included an intervention and control group; (3) had people with MS as study subjects; (4) had balance, walking ability, walking endurance, fatigue, or quality of life as the outcome measures. Exclusion criteria were: (1) non-English publications; (2) animal model publications; (3) review articles; and (4) conference articles.

### Data extraction

Two authors (L.D. and H.X.) independently performed the data extraction, mainly including: (1) study characteristics (surname of the first author, year of publication, and sample size); (2) intervention characteristics (intensity, duration, and frequency); (3) participant characteristics (gender, disease stage, and disease duration); (4) treatment effects [mean and standard deviation (SD) values reflecting changes in balance, walking ability, walking endurance, fatigue, and quality of life from baseline to post intervention].

### Methodological quality assessment

The methodological quality for the included studies was independently assessed by two authors (L.D. and H.X.) based on the Cochrane risk of bias tool (RoB2) (19) and Physiotherapy Evidence Database (PEDro) scale (20, 21). If there was disagreement between the two authors, a third author (LY) would join the discussion until the three reach a consensus. RoB2 was assessed mainly from seven items: random sequence generation (selection bias), allocation concealment (selection bias), blinding of participants and personnel (performance bias), blinding of outcome assessment (detection bias), incomplete outcome data (attrition bias), selective reporting (reporting bias), and other biases. PEDro scale is an 11-item scale used to evaluate the quality of the RCTs of the physical therapy studies, where studies scoring <4, 4–5, 6–8, and >9 points are considered poor quality, average, good, and excellent, respectively (21).

### Statistical analysis

We extracted the mean and SD values reflecting changes in timed up and go test (TUG), Berg balance scale (BBS), multiple sclerosis walking scale-12 (MSWS-12), 6-minute walk test (6MWT), fatigue severity scale (FSS), modified fatigue impact scale (MFIS), and multiple sclerosis quality of life-54 (MSQOL-54) from baseline to post-intervention from each study for pooling effects. Weighted mean difference (WMD) and 95% confidence interval (CI) were used to estimate the effects of exercise on balance, walking ability, walking endurance, fatigue, and quality of life in people with MS. For studies reporting standard error (SE) or 95% confidence interval (CI), SD was calculated using the previously described formula. Otherwise, PlotDigitizer online software (www.plotdigitizer.com) was used (22). The *I*^2^ static was used to assess heterogeneity, where *I*^2^ <25% indicates no significant heterogeneity, 25% <*I*^2^ <50% indicates low heterogeneity, 50% <*I*^2^ <75% indicates moderate heterogeneity, and *I*^2^ > 75% indicates high heterogeneity (23). If there was a high heterogeneity (*I*^2^ > 60%), meta-regression analysis, subgroup analysis, and sensitivity analysis were used to interpret the results (19).

For subgroup analyses, we examined the effects of intervention type (aerobic exercise, resistance exercise, and multicomponent exercise), participants' age (young, <45 years old; and middle-aged and older adult, ≥45 years old), and type of fatigue detection (FSS and MFIS) on fatigue and intervention type (aerobic exercise and multicomponent exercise) on quality of life in people with MS. Meta-regressions were conducted based on the participants' age, disease duration, duration of intervention, session duration, and weekly time. The analysis result, funnel plot, and forest plot were generated using RevMan 5.2 software. Statistical significance was considered for outcomes with a *P* < 0.05.

## Results

### Study selection

**Figure 1:**
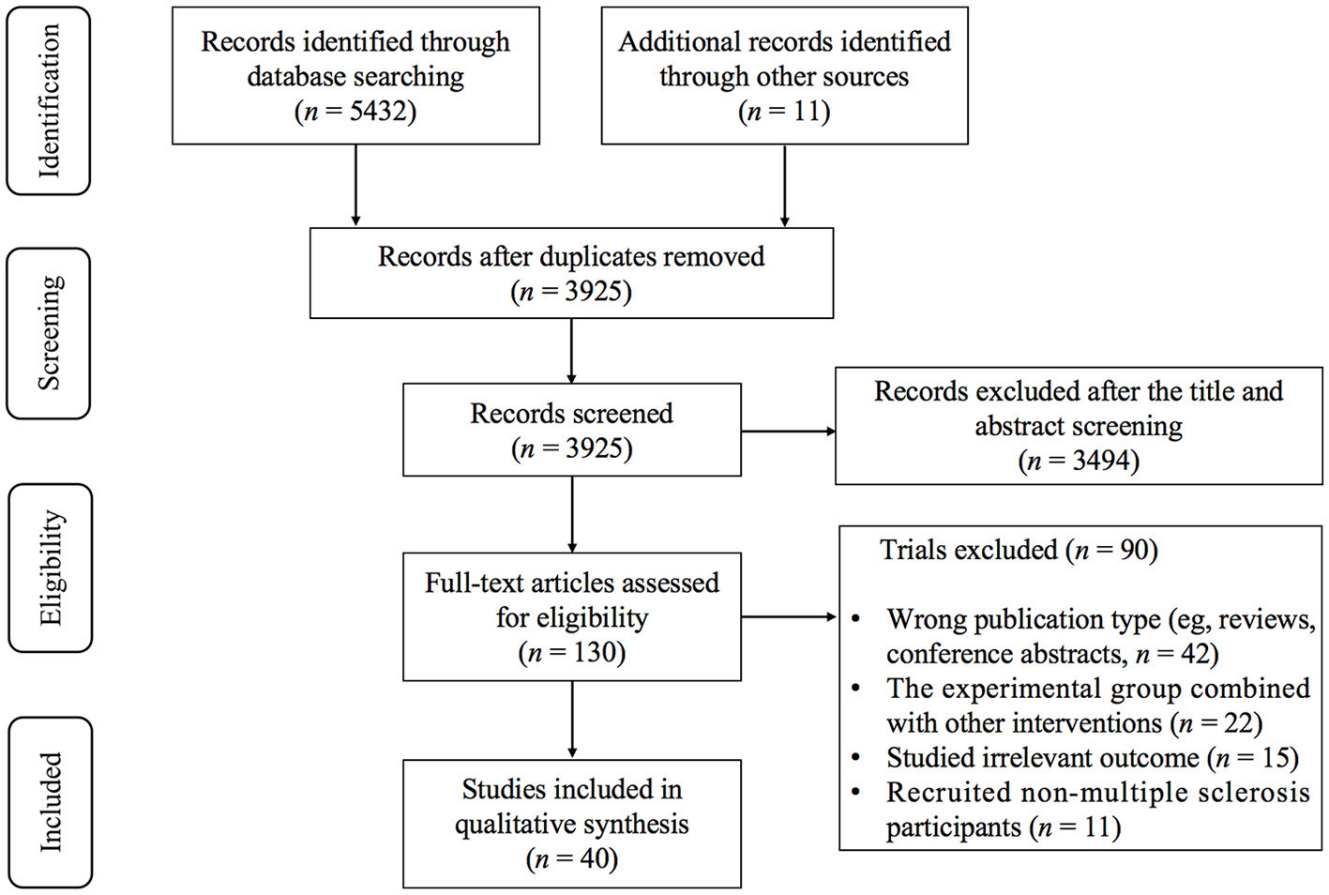
PRISMA flow diagram of the study selection process.

As shown in Figure 1, 5,432 records were initially identified from the databases and 11 records from other sources. Three thousand nine hundred and twenty-five studies remained after excluding duplicates and 130 potentially eligible studies remained after the title and abstract screening. Ninety studies were excluded by reading the full text: (1) wrong publication type (e.g., reviews, conference abstracts, *n* = 42); (2) the experimental group combined with other interventions (*n* = 22); (3) studied irrelevant outcome (*n* = 15); (4) recruited non-multiple sclerosis participants (*n* = 11). Finally, 40 studies (24–63) were considered eligible for systematic review and meta-analysis.

### Characteristics of the included studies

The main characteristics of participants and interventions were shown in Table 1. Among the included studies, there were 1,300 people with MS in the 56 exercise groups and 827 people with MS in the 40 control groups. Six studies involved women, 1 study involved men, and 30 studies involved both men and women. The mean age of the participants ranged from 16.3 to 61.6 years. Thirty-seven studies (24–28, 30–32, 34–49, 51–63) involved participants with mean age <60 years, and three studies (29, 33, 50) involved participants with mean age ≥60 year. Most interventions specified aerobic exercise (*n* = 16) (24–26, 30, 33, 34, 36, 38, 39, 45, 46, 51, 53, 56, 58, 63), balance training (*n* = 10) (29, 37, 43, 47, 50, 52, 54, 55, 59, 61), resistance exercise (*n* = 6) (27, 28, 32, 41, 44, 49), or other types of exercise [such as multicomponent training (*n* = 5) (31, 35, 42, 48, 62) water sports (*n* = 2) (40, 57); interval training (*n* = 1) (60)]. Of the 40 studies, 26 studies provided data for balance, which was tested by BBS (20 studies) (24, 29, 34, 36–40, 42–44, 46, 49, 50, 52–54, 58, 59, 61) and TUG (17 studies) (25–27, 33, 34, 37, 38, 42–44, 50, 51, 54–56, 59, 61, 62). In addition, 17 studies provided data for gait, which was tested by MSWS-12 (walking ability, eight studies) (29, 30, 34, 47, 50, 55, 60, 61) and 6MWT (walking endurance, 14 studies) (25, 28, 30, 39, 40, 42, 45, 48, 51, 53, 54, 56, 60, 61). Furthermore, 17 studies provided data for fatigue, which was tested by FSS (nine studies) (24, 26, 28, 42, 44, 51–54) and MFIS (eight studies) (30, 32, 33, 39, 41, 43, 50, 57). Moreover, six studies provided data for quality of life (24, 31, 45, 48, 57, 63), which was tested by MSQOL-54.

**Table 1:** Characteristics of the studies included in this meta-analysis.

**References**	**Sample size**	**Sex**	**Age (y)**	**EDSS**	**Disease duration (y)**	**Intervention**	**Details of interventions**	**Outcome measures**
Ahmadi et al. (24)	IG = 11	11 W	IG: 32.3 (8.7)	IG: 2.0 (1.1)	IG: 4.7 (5.6)	Yoga	8 weeks, 60–70 min, each position for 10–30 s, group rest for 30–60 s, 3 times/week	FFS and MSQOL-54
	CG = 10	10 W	CG: 36.7 (9.3)	CG: 2.3 (1.3)	CG: 5.0 (3.1)	Usual care		
Androwis et al. (25)	IG = 6	3 M and 3 W	IG: 46.5 (5.2)	NR	NR	Walking	4 weeks, 30 min, 2 times/week	TUG and 6MWT
	CG = 4	1 M and 3 W	CG: 55.0 (9.6)	NR	NR	Rehabilitation nursing		
Cakt et al. (26)	IG = 14	5 M and 9 W	IG: 36.4 (10.5)	NR	IG: 9.2 (5.0)	Bicycle	8 weeks, 30–35 min, 30–40 W low resistance, twice/week	TUG and FFS
	IG = 10	2 M and 8 W	IG: 43.0 (10.2)	NR	IG: 6.2 (2.2)	Balance training	8 weeks, 30–35 min, 2 times/week	
	CG = 9	3 M and 6 W	CG: 35.5 (10.9)	NR	CG: 6.6 (2.4)	Usual care		
Andreu-Caravaca et al. (27)	IG = 18	10 M and 8 W	IG: 44.9 (10.6)	IG: 3.2 (1.7)	NR	Strength training	10 weeks, 40% 1 RM, 3 times per week	TUG
	CG = 12	5 M and 7 W	CG: 48.4 (10.2)	CG: 3.3 (1.3)	NR	Usual care		
Andreu-Caravaca et al. (28)	IG = 18	10 M and 8 W	IG: 44.9 (10.6)	IG: 3.2 (1.7)	NR	Strength training	10 weeks, 40% 1 RM, 3 times per week	6MWT and FSS
	CG = 12	5 M and 7 W	CG: 48.4 (10.2)	CG: 3.3 (1.3)	NR	Usual care		
Carling et al. (29)	IG = 25	6 M and 19 W	IG: 61.6 (11.3)	IG: 6.2 (0.5)	NR	Balance training	7 weeks, 60 min, 2 times/week	BBS, TUG, and MSWS-12
	CG = 26	10 M and 16 W	CG: 54.7 (8.2)	CG: 6.1 (0.5)	NR	Usual care		
Langeskov-Christensen et al. (30)	IG = 43	17 M and 26 W	IG: 44.0 (9.5)	IG: 2.7(1.4)	IG: 10.9 (7.9)	Aerobic training	24 weeks, 30–60 min, 65–95% maximum heart rate, twice/week	MFIS, FSS, 6MWT, and MSWS-12
	CG = 43	17 M and 26 W	CG: 45.6 (9.3)	CG: 2.8(1.6)	CG: 8.6 (6.0)	Usual care		
Correale et al. (31)	IG = 14	14 W	IG: 45.4 (7.2)	NR	NR	Combination training	12 weeks, 45–60 min, 50–70% reserve heart rate, 2 times/week	MFIS and MSQOL-54
	CG = 9	9 W	CG: 48.3 (6.1)	NR	NR	Usual care		
Dodd et al. (32)	IG = 36	10 M and 26 W	IG: 47.7 (10.8)	NR	NR	Strength training	10 weeks, 45 min, 2 sets per action, 10–12 times, 2 times per week	MFIS
	CG = 35	9 M and 26 W	CG: 50.4 (9.6)	NR	NR	Usual care		
Fleming et al. (33)	IG = 29	29 W	IG: 45.3 (8.6)	NR	NR	Pilates	8 weeks, repeat actions 4–10 times, 2 times/week	MFIS
	CG = 34	34 W	CG: 48.2 (9.8)	NR	NR	Usual care		
Forsberg et al. (34)	IG = 35	28 M and 7 W	IG: 52.0 (10)	NR	IG: 15.0 (9.0)	Core training	7 weeks, 50–60 min, 2 times/week	BBS, TUG, and MSWS-12
	CG = 38	31 M and 7 W	CG: 56.3 (11)	NR	CG: 16.0 (11.0)	Usual care		
Garrett et al. (35)	IG = 63	13 M and 50 W	IG: 51.7 (10)	NR	IG: 9.8 (7.0)	Physical therapy	10 weeks, 12 actions per action, with/2–5% increase in load during easy times, 60 min per week	MFIS and 6MWT
	IG = 67	22 M and 45 W	IG: 50.3 (10)	NR	IG: 10.5 (6.9)	Combination training	10 weeks, 60 min per week	
	IG = 63	19 M and 44 W	IG: 49.6 (10)	NR	IG: 11.6 (8.0)	Yoga	10 weeks, action duration 30–90 s, 60 min per week	
	CG = 49	6 M and 43 W	CG: 48.8 (11)	NR	CG: 10.6 (8.2)	Usual care		
Gervasoni et al. (36)	IG = 15	NR	IG: 49.6 (9.4)	NR	IG: 14.5 (9.7)	Treadmill	2 weeks, 45 min, 11–12 RPE intensity, completed 10–12 times in 2 weeks	BBS and FSS
	CG = 15	NR	CG: 45.7 (8.9)	NR	CG: 15.5 (10.3)	Usual care		
Eftekharsadat et al. (37)	IG = 15	5 M and 10 W	IG: 33.4 (8.1)	NR	IG: 5.8 (3.9)	Stability training	12 weeks, 20 min, 2 times/week	BBS and TUG
	CG = 15	3 M and 12 W	CG: 37.0 (8.3)	NR	CG: 8.3 (4.3)	Usual care		
Gheitasi et al. (38)	IG = 15	15 M	IG: 30.6 (5.3)	IG: 4.6 (1.6)	IG: 5.5 (1.1)	Pilates	12 weeks, 60 min, 3 times/week	TUG
	CG = 15	15 M	CG: 32.1 (6.3)	CG: 4.5 (1.1)	CG: 4.0 (1.0)	Usual care		
Hogan et al. (39)	IG = 35	15 M and 20 W	IG: 52.0 (11.0)	NR	IG: 13.0 (8.0)	Personal balance training	10 weeks, 60 min per week	MFIS, BBS, and 6MWT
	IG = 48	18 M and 30 W	IG: 57.0 (10.0)	NR	IG: 18.0 (9.0)	Group balance training	10 weeks, 60 min per week	
	IG = 13	5 M and 8 W	IG: 58.0 (8.0)	NR	IG: 15.0 (8.0)	Yoga	10 weeks, 60 min per week	
	CG = 15	2 M and 13 W	CG: 49.0 (6.0)	NR	CG: 10.0 (3.0)	Usual care		
Kargarfard et al. (40)	IG = 17	NR	IG: 36.5 (9.0)	IG: 3.4 (1.1)	IG: 6.4 (2.3)	Water sports	8 weeks, 30–40 min	6MWT, BBS, MFIS, and MSQOL-54
	CG = 15	NR	CG: 36.2 (7.4)	CG: 3.7 (1.0)	CG: 6.1 (2.0)	Usual care		
Learmont et al. (42)	IG = 20	5 M and 15 W	IG: 51.4 (8.06)	IG: 6.1 (0.4)	IG: 13.4 (6.4)	Aerobic, resistance, and balance training	12 weeks, 45–60 min	6MWT, BBS, TUG, and FSS
	CG = 12	4 M and 8 W	CG: 51.8 (8.0)	CG: 5.8 (0.5)	CG: 12.6 (8.1)	Usual care		
Najafi et al. (43)	IG = 28	28 W	IG: 38.4 (4.6)	IG: 2.5 (1.2)	NR	Stability training	8 weeks, 60–80 min, 3 times/week	TUG and BBS
	CG = 28	28 W	CG: 36.4 (3.5)	CG: 2.4 (0.8)	NR	Usual care		
Negahban et al. (44)	IG = 12	NR	IG: 36.7 (6.7)	IG: 3.5 (1.1)	IG: 8.5 (6.8)	Strength training	5 weeks, 30 min, 3 times/week	FSS, BBS, and TUG
	CG = 12	NR	CG: 36.8 (8.7)	CG: 3.8 (1.4)	CG: 7.2 (2.9)	Usual care		
Ozkul et al. (45)	IG = 17	4 M and 13 W	IG: 35.9 (9.7)	IG: 1.5 (0.7)	IG: 7.2 (6.1)	Pilates	8 weeks, 50–60 min, 60–80 maximum heart rate, 3 times per week	6MWT and MSQOL-54
	CG = 17	4 M and 13 W	CG: 36.8 (9.0)	CG: 1.7 (0.9)	CG: 5.7 (4.9)	Usual care		
Pan et al. (46)	IG = 30	8 M and 22 W	IG: 42.2 (5.1)	IG: 3.0 (0.7)	IG: 6.2 (2.3)	Baduanjin	24 weeks, 60 min per day	BBS
	IG = 30	9 M and 21 W	IG: 40.9 (4.8)	IG: 2.8 (0.9)	IG: 5.2 (2.0)	Yoga	24 weeks, 60 min per day	
	CG = 20	6 M and 14 W	CG: 42.3 (4.5)	CG: 2.9 (0.8)	CG: 5.4 (2.8)	Usual care		
Robinson et al. (47)	IG = 20	6 M and 14 W	IG: 52.6 (6.1)	NR	NR	Balance game	4 weeks, 40–60 min, 2 times/week	FSS and MSWS-12
	IG = 19	7 M and 12 W	IG: 53.9 (6.5)	NR	NR	Balance training	4 weeks, 40–60 min, 2 times/week	
	CG = 17	5 M and 12 W	CG: 51.9 (4.7)	NR	NR	Usual care		
Romberg et al. (48)	IG = 47	17 M and 30 W	IG: 43.8 (6.3)	NR	IG: 6.0 (6.5)	Combination training	26 weeks, 3–4 times/week	MSQOL-54
	CG = 48	17 M and 31 W	CG: 43.9 (7.1)	NR	CG: 5.5 (6.4)	Usual care		
Sokhangu et al. (49)	IG = 10	10 W	IG: 38.7 (7.2)	IG: 1.8 (0.7)	IG: 4.2 (2.1)	Strength training	8 weeks, 60 min, 8–15 times per action, 3 times per week	BBS
	CG = 10	10 W	CG: 40.1 (5.6)	CG: 1.9 (0.7)	CG: 4.4 (2.0)	Usual care		
Sosnoff et al. (50)	IG = 13	3 M and 10 W	IG: 60.1 (6.3)	IG: 5.5 (2.5)	IG: 13.9 (6.7)	Balance training	12 weeks, 1–3 groups, 8–12 times, 45–60 min	TUG, 6MWT, BBS, and MSWS-12
	CG = 14	3 M and 11 W	CG: 60.1 (6.0)	5.5 (3.5)	17.7 (11.3)	Usual care		
Straudi et al. (51)	IG = 8	4 M and 4 W	IG: 49.6 (12.0)	IG: 5.8 (0.8)	IG: 17.1 (12.0)	Gait practice	6 weeks, 30 min, 2 times/week	6MWT and TUG
	CG = 8	1 M and 7 W	CG: 60.0 (8.8)	CG: 5.7 (0.7)	CG: 18.6 (10.8)	Usual care		
Tarakci et al. (52)	IG = 51	17 M and 34 W	IG: 41.5 (9.4)	IG: 4.9 (1.4)	IG: 9.0 (4.7)	Balance training	12 weeks, 60 min, 3 times/week	BBS and FSS
	CG = 48	18 M and 30 W	CG: 39.7 (11.2)	CG: 4.2 (1.4)	CG: 8.4 (5.4)	Usual care		
Tollár et al. (53)	IG = 14	2 M and 12 W	IG: 48.2 (5.5)	NR	IG: 12.1 (2.7)	Agility training	5 weeks, 60 min, 5 times/week	BBS and 6MWT
	IG = 14	2 M and 12 W	IG: 46.9 (6.5)	NR	IG: 13.6 (4.1)	Balance training	5 weeks, 60 min, 5 times/week	
	IG = 14	2 M and 12 W	IG: 48.1 (5.7)	NR	IG: 13.2 (4.4)	Bicycle	5 weeks, 60 min, 5 times/week	
	CG = 12	1 M and 11 W	CG: 44.4 (6.8)	NR	CG: 14.0 (4.11)	Usual care		
Grubić Kezele et al. (41)	IG = 13	5 M and 8 W	IG: 50.0 (9.3)	IG: 3.8 (1.8)	NR	Strength training	8 weeks, 60 min, 2 times/week	MFIS
	CG = 11	5 M and 6 W	CG: 53.8 (13.8)	CG: 4.0 (2.0)	NR	Usual care		
Yazgan et al. (54)	IG = 15	2 M and 13 W	IG: 47.5 (10.5)	IG: 4.2 (1.4)	IG: 12.1 (6.6)	Balance game	8 weeks, 60 min, 2 times/week	BBS, TUG, and FSS
	IG = 12	12 W	IG: 43.1 (8.7)	IG: 3.8 (1.5)	IG: 14.9 (6.6)	Balance training	8 weeks, 60 min, 2 times/week	
	CG = 15	2 M and 13 W	CG: 40.7 (8.8)	CG: 4.1 (1.3)	CG: 11.1 (5.1)	Usual care		
Young et al. (56)	IG = 27	5 M and 22 W	IG: 49.7 (9.4)	NR	IG: 13.6 (8.3)	Strength training	12 weeks, 60 min, 3 times/week	TUG and 6MWT
	IG = 26	6 M and 20 W	IG: 48.4 (10.0)	NR	IG: 11.0 (5.6)	Yoga	12 weeks, 60 min, 3 times/week	
	CG = 28	4 M and 24 W	CG: 47.3 (10.3)	NR	CG: 13.4 (8.5)	Usual care		
Kargarfard et al. (57)	IG = 10	NR	IG: 33.7 (8.6)	IG: 2.9 (0.9)	IG: 4.9 (2.3)	Aquatic exercise	8 weeks, 60 minutes, 3 times/week	MFIS
	CG = 11	NR	CG: 31.6 (7.7)	CG: 3.0 (0.7)	CG: 4.6 (1.9)	Usual care		
Nilsagård et al. (55)	IG = 41	10 M and 31 W	IG: 50.0 (11.5)	NR	IG: 12.5 (8.0)	Balance training	6 weeks, 30 min, 2 times/week	TUG and MSWS-12
	CG = 39	10 M and 29 W	CG: 49.4 (11.1)	NR	CG: 12.2 (9.2)	Usual care		
Ahadi et al. (63)	IG = 10	10 W	IG: 50.0 (11.5)	NR	NR	Running	8 weeks, 30 min, 3 times/week	MSQOL-54
	IG = 11	11 W	IG: 50.0 (11.5)	NR	NR	Yoga	8 weeks, 30 min, 3 times/week	
	CG = 10	10 W	CG: 49.4 (11.1)	NR	NR	Usual care		
Abadi Marand et al. (61)	IG = 32	17 M and 15 W	IG: 40.4 (6.0)	IG: 4.1 (1.1)	IG: 14.4 (5.2)	Balance training	5 weeks, 60–70 min, 3 times/week	BBS, TUG, and MSWS-12
	CG = 32	18 M and 14 W	CG: 40.7 (6.2)	CG: 3.8 (1.0)	CG: 12.8 (5.9)	Usual care		
Monjezi et al. (59)	IG = 17	3 M and 14 W	IG: 38.1 (9.5)	IG: 4.8 (1.0)	IG: 9.7 (6.3)	Balance Training	4 weeks, 20 min, 3 times/week	BBS and TUG
	CG = 17	3 M and 14 W	CG: 35.1 (8.0)	CG: 4.6 (0.7)	CG: 8.9 (5.2)	Usual care		
Vural et al. (62)	IG = 10	2 M and 8 W	IG: 16.3 (1.6)	IG: 1.2 (0.8)	IG: 2.3 (1.2)	Combination training	8 weeks, 60 min, 2 times/week	FSS, TUG, and 6MWT
	IG = 10	2 M and 8 W	IG: 16.3 (1.6)	IG: 1.2 (0.8)	IG: 2.3 (1.2)	Combination training	32 weeks, 60 min, 2 times/week	
	CG = 10	1 M and 9 W	CG: 17.4 (1.8)	CG: 1.7 (0.8)	CG: 2.3 (1.7)	Usual care		
Lysogorska et al. (58)	IG = 15	5 M and 10 W	IG: 39.0 (10.4)	NR	IG: 12.6 (8.4)	Yoga	12 weeks, 60–75 min, 2 times/week	BBS and 6MWT
	IG = 9	9 W	IG: 46.1 (10.3)	NR	IG: 18.1 (12.3)	Combination training	12 weeks, 60–75 min, 2 times/week	
	CG = 12	1 M and 11 W	CG: 46.2 (10.4)	NR	CG: 18.5 (7.9)	Usual care		
Riemenschneider et al. (60)	IG = 42	13 M and 29 W	IG: 37.3 (10.1)	IG: 1.4 (0.9)	IG: 0.9 (0.6)	Interval training	24 weeks, 30–60 min, 2 times/week	6MWT and MSWS-12
	IG = 42	13 M and 29 W	IG: 37.3 (10.1)	IG: 1.4 (0.9)	IG: 0.9 (0.6)	Interval training	48 weeks, 30–60 min, 2 times/week	
	CG = 42	8 M and 34 W	CG: 37.4 (9.7)	CG: 1.8 (1.1)	CG: 0.9 (0.6)	Usual care		

IG, intervention group; CG, control group; M, male; W, woman; NR, no report; TUG, timed up and go test; BBS, Berg balance scale; 6MWT, 6-minute walk test; MSIS-12, The 12-Item MS Walking Scale; FSS, Fatigue Severity Scale; MFIS, modified fatigue impact scale; MSQOL-54, Quality of Life−54 Questionnaire.

Data were presented as mean (standard deviation).

### Meta-analysis results

#### Effects of exercise on balance in people with MS

**Figure 2:**
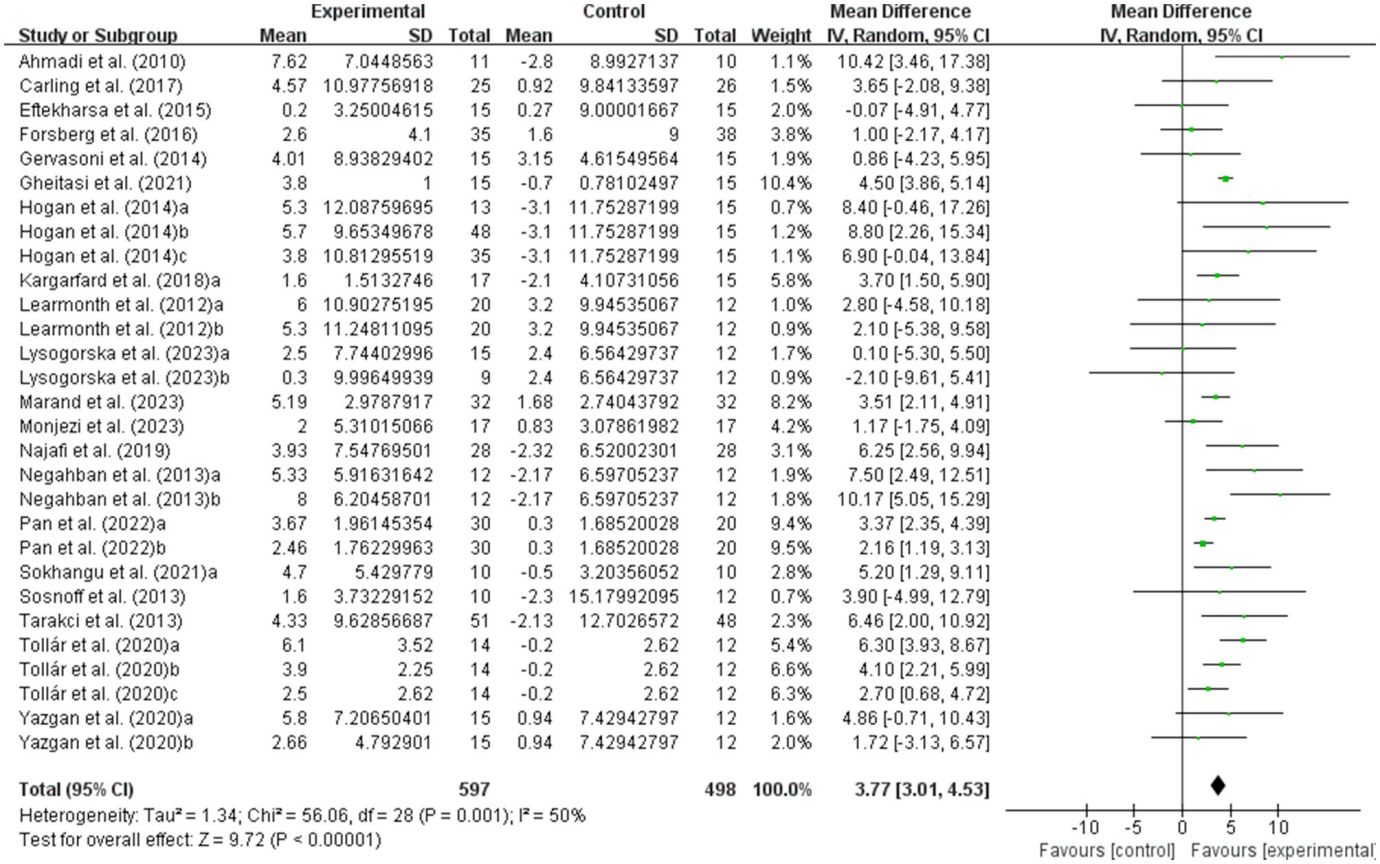
Meta-analysis results of the effects of exercise on Berg balance scale (BBS) in people with MS. Exercise had a significant effect on improving BBS in people with MS (*P* < 0.00001).

**Figure 3:**
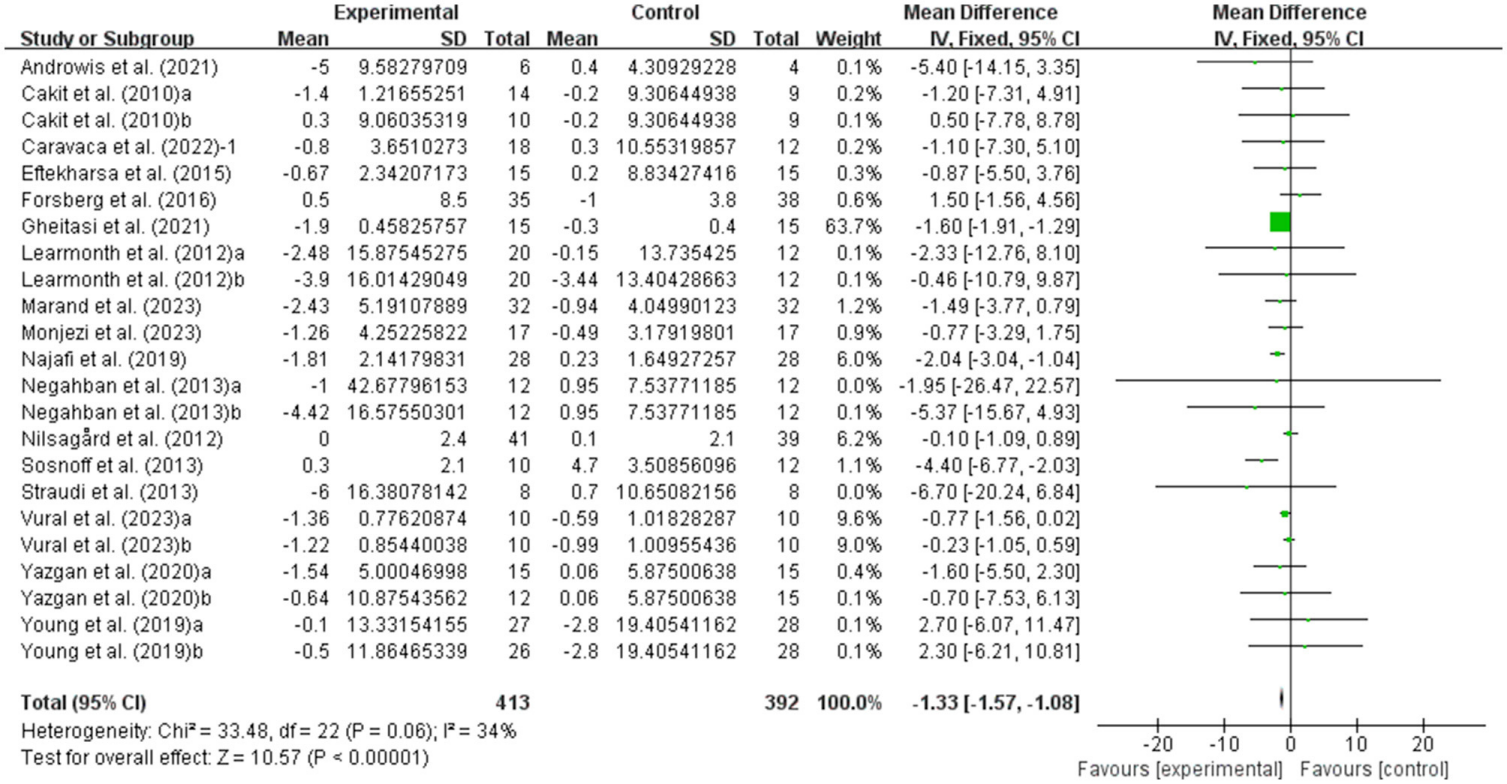
Meta-analysis results of the effect of exercise on timed up and go test (TUG) in people with MS. Exercise had a significant effect on improving TUG in people with MS (*P* < 0.00001).

The balance of people with MS was detected by BBS and TUG, with 20 studies providing BBS data and 20 studies providing TUG data. Our results showed that exercise had a significant effect on improving BBS (WMD, 3.77; 95% CI, 3.01 to 4.53, *P* < 0.00001, *I*^2^ = 50%, Figure 2) and TUG (WMD, −1.33; 95% CI, −1.57 to −1.08, *P* < 0.00001, *I*^2^ = 34%, Figure 3) in people with MS.

#### Effects of exercise on walking ability and walking endurance in people with MS

**Figure 4:**
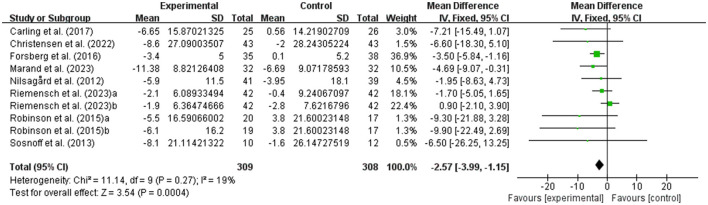
Meta-analysis results of the effects of exercise on walking ability in people with MS. Exercise had a significant effect on improving walking ability in people with MS (*P* = 0.0004).

**Figure 5:**
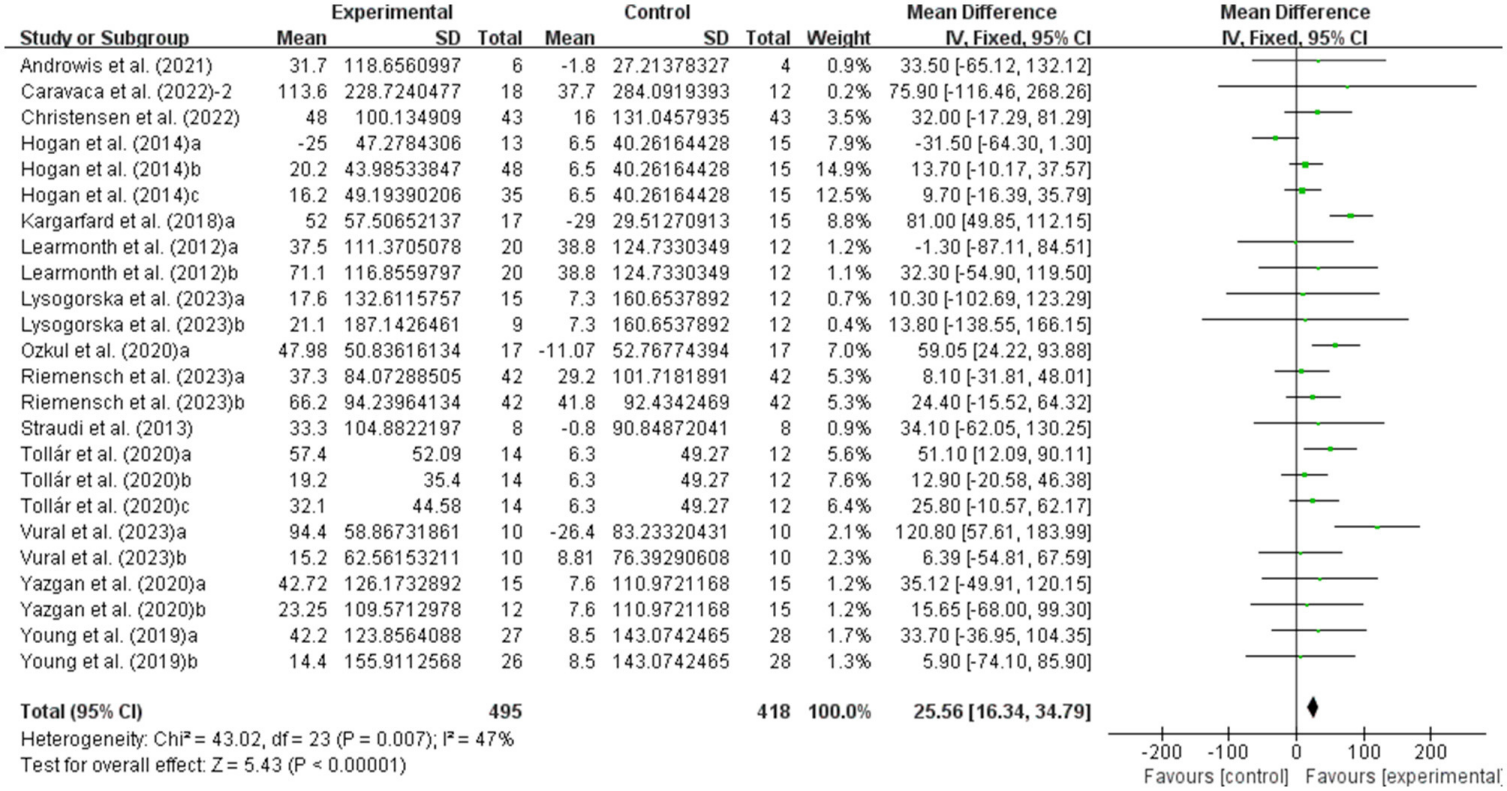
Meta-analysis results of the effects of exercise on walking endurance in people with MS. Exercise had a significant effect on improving walking endurance in people with MS (*P* < 0.00001).

**Figure 6:**
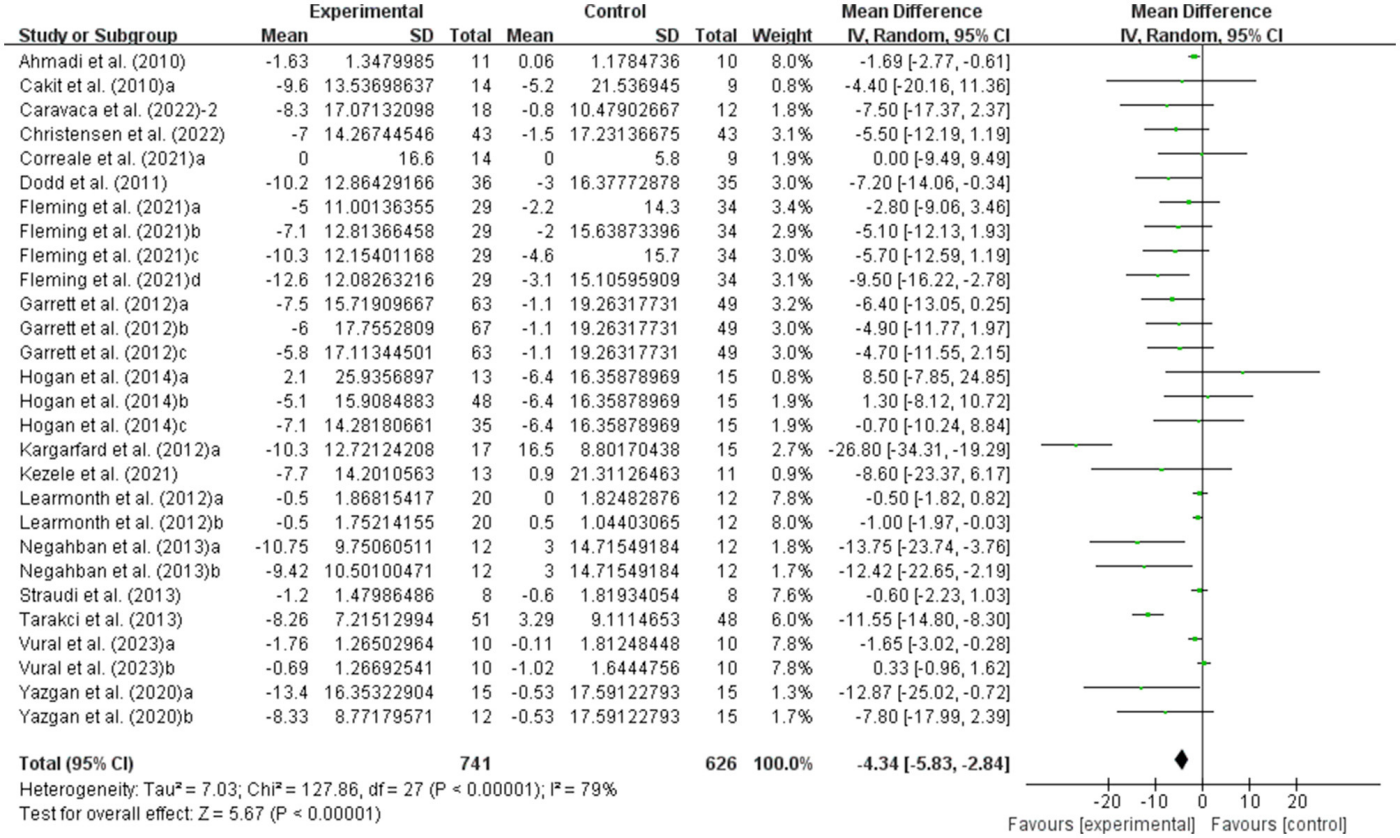
Results of the effects of exercise on fatigue in people with MS. Exercise had a significant effect on improving fatigue in people with MS (*P* < 0.00001).

MSWS-12 was used to test walking ability and 6MWT was used to test walking endurance of people with MS. It was found that exercise had a significant effect on improving MSWS-12 (WMD, −2.57; 95% CI, −3.99 to −1.15, *P* = 0.0004, *I*^2^ = 19%, Figure 4) and 6MWT (WMD, 25.56; 95% CI, 16.34 to 34.79, *P* < 0.00001, *I*^2^ = 47%, Figure 5) in people with MS.

#### Effects of exercise on fatigue in people with MS

The fatigue of people with MS was detected by FSS and MFIS. As shown in Figure 6, exercise had a significant effect on improving fatigue in people with MS (WMD, −4.34; 95% CI, −5.83 to −2.84, *P* < 0.00001, *I*^2^ = 79%).

#### Effects of exercise on quality of life in people with MS

**Figure 7:**
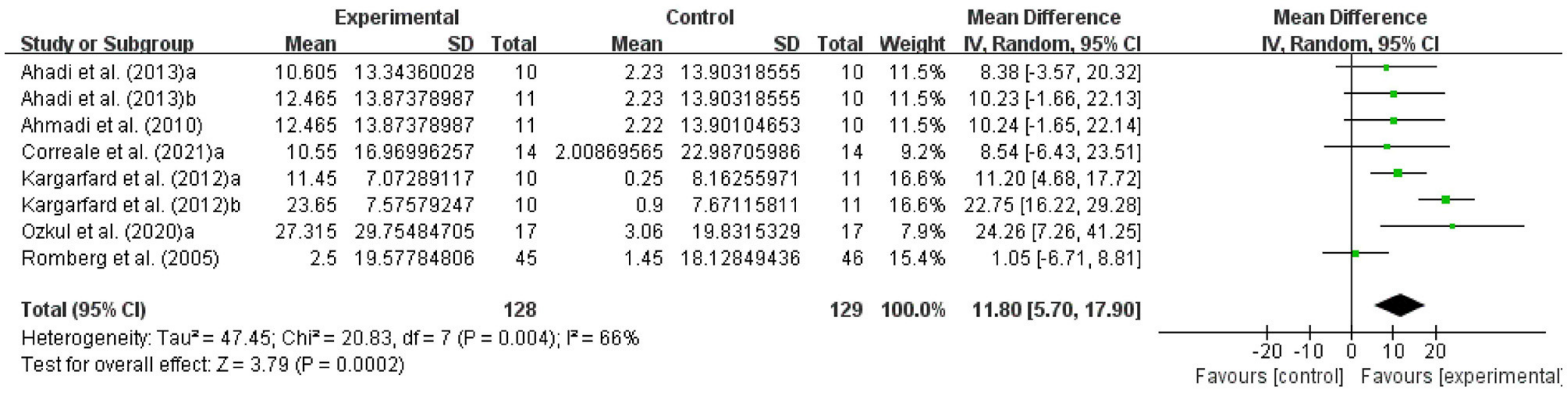
Results of the effects of exercise on quality of life in people with MS. Exercise had a significant effect on improving quality of life in people with MS (*P* = 0.0002).

The fatigue of people with MS was detected by MSQOL-54. As shown in Figure 7, exercise had a significant effect on improving MSQOL-54 in people with MS (WMD, 11.80; 95% CI, 5.70 to 17.90, *P* = 0.0002, *I*^2^ = 66%).

Our meta-analysis results showed high heterogeneity in fatigue (*I*^2^ = 78%) and quality of life (*I*^2^ = 66%), to explain the heterogeneity between included studies and find modifiable factors of exercise, meta-regression analysis, subgroup analysis, and sensitivity analysis were further performed.

### Meta-regression analysis

Meta-regression analyses were performed on intervention characteristics (duration of intervention, weekly time, and session duration) and participant characteristics (age and disease duration). There was no significant association between age (*P* = 0.782), duration of intervention (*P* = 0.124), weekly time (*P* = 0.730), session duration (*P* = 0.124), or disease duration (*P* = 0.559) and fatigue (Supplementary Figure 1). In addition, no significant associations were observed between duration of intervention (*P* = 0.086), weekly time (*P* = 0.583), session duration (*P* = 0.878), age (*P* = 0.172), or disease duration (*P* = 0.289) and quality of life (Supplementary Figure 2).

### Subgroup analysis

#### Fatigue

We conducted three different subgroup analyses by participants' age, type of fatigue detection, and type of intervention. Subgroup analysis indicated that a younger age was associated with larger improvement in fatigue (young, age <45, WMD, −6.67; 95% CI, −9.57 to −3.60, *P* < 0.0001, *I*^2^ = 91%; middle-aged and older adult, age ≥ 45, WMD, −1.76; 95% CI, −3.29 to −0.24, *P* = 0.02, *I*^2^ = 22%, Supplementary Figure 3).

Stratifying the analysis by type of fatigue detection, the improvement in fatigue scores remained significant in FSS (WMD, −2.75; 95% CI, −4.27 to −1.24, *P* = 0.0004, *I*^2^ = 81%) and MFIS (WMD, −5.84; 95% CI, −9.28 to −2.40, *P* = 0.0009, *I*^2^ = 65%, Supplementary Figure 4).

In addition, aerobic exercise (WMD, −7.07; 95% CI, −11.25 to −2.88, *P* = 0.0009, *I*^2^ = 81%), resistance exercise (WMD, −8.03; 95% CI, −11.84 to −4.22, *P* < 0.0001, *I*^2^ = 0%), and multicomponent training (WMD, −2.54; 95% CI, −4.44 to −0.65, *P* = 0.009, *I*^2^ = 80%) were effective in improving fatigue in people with MS, with resistance exercise being the most effective intervention type (Supplementary Figure 5).

#### Quality of life

We conducted a subgroup analysis by type of intervention. Aerobic exercise (WMD, 11.68; 95% CI, 5.31 to 18.05, *P* = 0.0003, *I*^2^ = 0%) and multicomponent training (WMD, 7.28; 95% CI, 2.77 to 11.79, *P* = 0.002, *I*^2^ = 24%) were effective in improving quality of life in people with MS, with aerobic exercise being the most effective intervention type (Supplementary Figure 6).

### Sensitivity analysis

Sensitivity analyses showed that there is no change in the direction or level of compatibility of the overall effect of exercise on fatigue (Supplementary Figure 7) and quality of life (Supplementary Figure 8) in people with MS when any of the included studies are omitted.

### Risk of bias

The quality of included studies was assessed by the Cochrane Collaboration tool in terms of selection bias, performance bias, attrition bias, reporting bias, detection bias, and other bias (Supplementary Figure 9). The results of PEDro scale showed that of the 40 included studies, 39 were of good quality and one was of fair quality (Supplementary Table 2).

### Publication bias

Possible publication bias was assessed by examining the funnel plot (Supplementary Figure 10). Visual inspection of the funnel plot suggested the absence of funnel plot asymmetry. The results of the egger's test indicated that the small sample size studies were not enough to affect the final results (TUG, *P* = 0.575; BBS, *P* = 0.705; 6MWT, *P* = 0.586; MSWS-12, *P* = 0.137; quality of life, *P* = 0.791; Supplementary Table 3), with the exception of fatigue (*P* = 0.002). Therefore, we performed the Dsuval and Tweedie's trim and fill procedure, and the results indicated that no evidence of publication bias was found for fatigue.

## Discussion

In this systematic review and meta-analysis, exercise significantly improved balance, walking ability, walking endurance, fatigue, and quality of life in people with MS. Subgroup analyses showed that a younger age was associated with larger improvement in fatigue. In addition, resistance exercise and aerobic exercise were the most effective interventions for improving fatigue and quality of life, respectively.

Loss of balance and walking ability are two of the primary impairments of MS that leads to increased fatigue perception and disease severity, and loss of autonomy (13). Imbalance, gait dysfunction and falls are common in people with MS, with the overwhelming majority having abnormal postural control and gait even early in the course of the disease. It has been reported that 50–80% people with MS have balance and gait dysfunction and over 50% fall at least once each year (64). Exercise has been shown to improve physical function and psychological rehabilitation in people with MS, and to help reduce the risk of falls (65, 66). Our study showed that exercise significantly improved balance function (TUG and BBS) in people with MS, which was consistent with a previous study (13), showing that the combination of resistance and aerobic exercise training is effective in improving balance in people with MS and supports functional and psychological therapeutic effects through exercise. In addition, a meta-analysis showed that yoga was the best intervention to improve static and dynamic balance, and aquatic training was the best intervention to improve walking ability in people with MS (67). The mechanisms by which exercise improves balance may be that exercise improves neurological control of muscles, increases unconscious deliberate muscle responses to dynamic joint stabilization signals, and enhances core area muscle strength to strengthen body stability.

Our results showed that exercise significantly improved walking ability (MSWS-12) and walking endurance (6MWT) in people with MS, which was in agreement with previous studies, showing that aerobic exercise, aquatic exercise, virtual reality training, and assisted gait training significantly improved walking ability (67–69), as well as that Pilates, aerobic exercise, resistance exercise, high-intensity training, and intermittent walking training significantly improved walking endurance in people with MS (28, 68, 70–72). Furthermore, fast-velocity concentric resistance training may have a greater effect on walking endurance with greater neural adaptations in a shorter period of time (28). A meta-analysis showed that walking training programs significantly improved functional ability (mobility, walking endurance, and gait speed), possibly due to improved walking economy (68). The mechanisms by which exercise improves walking ability and walking endurance in people with MS may be improvements in maximal oxygen uptake, muscular strength, and fitness. The increase in muscle strength is due to improved firing and synchronization of motor units and improved synergistic coordination of agonists and antagonists (73). Moreover, another mechanism may be increased bilateral symmetry, which reduces the amount of time the lower limbs are supported on the ground (74).

Early fatigue in people with MS presents with common symptoms such as decreased endurance and muscle strength (75). Statistically, fatigue affects approximately two-thirds of people with MS (76). Current evidence suggests that pharmacological interventions are largely ineffective and that exercise significantly reduces fatigue in people with MS (77, 78). Our results showed that exercise significantly improved fatigue in people with MS, which was consistent with the results of Taul-Madsen et al. (79), showing that aerobic exercise is effective in reducing perceived fatigue in people with MS. The mechanism by which exercise improves fatigue may be an improvement in cardiorespiratory fitness, which increases available energy reserve and reduces fatigue. In addition, exercise may induce upregulation of neuroendocrine growth factor secretion, which increases neuronal plasticity and thus may improve compensatory cortical activation (80, 81). Furthermore, exercise-induced upregulation of anti-inflammatory cytokines may have beneficial effects on fatigue (82–84).

Resistance exercise has been reported to be an effective intervention to ameliorate physical and generalized fatigue and result in significant changes in muscle strength and postural stability (85). Subgroup analysis showed that aerobic exercise, resistance exercise, and multicomponent training were effective in improving fatigue in people with MS, with resistance exercise being the most effective intervention type, which may be due to the fact that resistance exercise is well-tolerated in people with MS, restores the ability to respond quickly to stimuli, and improves autonomy when walking (13). Previous studies have shown that motor and cognitive function deteriorate with age in adult people with MS and that older people with MS exhibit worse cognitive performance (86–89). Therefore, we conducted a subgroup analysis based on the participants' age and the results showed that a younger age was associated with larger improvement in fatigue. Horton et al. (90) showed that with age, people with MS develop a sedentary lifestyle, which increases the risk of secondary disease. Although exercise is an effective therapy, dyskinesia is common in older adult patients. Increased fatigue is a severe barrier when exercise energy expenditure is relatively high, and older patients can lose confidence in their ability to exercise and may feel at risk of injury, especially when exercise equipment is involved (91–94).

In addition, exercise significantly improved the quality of life in people with MS, which was consistent with a previous study, showing that exercise seems to be the most effective way to improve the quality of life in people with MS by increasing strength and balance, thereby reducing the risk of falls (94). Previous studies have shown that multicomponent training is well-tolerated and can effective in improving the quality of life in people with MS (13), and that group exercise is an effective intervention for people with MS to cope with fatigue, with the Baduanjin playing a more prominent role in improving the quality of life through respiration and psychology (46). Improvements in quality of life may be related to exercise-induced increases in fitness, mobility, balance, muscle strength, and sleep quality (53, 95). Subgroup analysis showed that aerobic exercise and multicomponent training were effective in improving quality of life, with aerobic exercise being the most effective intervention type, which was in agreement with previous studies, showing that aerobic exercise increases aerobic capacity and improves physical and mental health, thereby enhancing functional independence and fatigue resistance in people with MS (96). In addition, aerobic exercise may stimulate the activity of the sympathetic nervous system and activate the activity of the parasympathetic nervous system, which leads to the release of acetylcholine, resulting in a sedative effect (97).

### Strengths and limitations of this systematic review

In this systematic review and meta-analysis, we included studies on the effect of exercise on balance, walking ability, walking endurance, fatigue, and quality of life in people with MS, and excluded studies where participants in the control group received exercise interventions, which can better reflect the effect of exercise interventions. Our findings provide an alternative treatment strategy for people with MS, clinically recommending engagement in resistance exercise and aerobic exercise, respectively, to alleviate fatigue and enhance quality of life.

However, this study has some limitations that should be noted. First, the heterogeneity between each of the original studies is unavoidable (the proportion of male and female participants from different regions, the age of subjects, etc.), which may affect the scientific validity of the meta-analysis. Second, many of the included studies had small sample sizes, which may have had some impact on the results. Finally, it was not possible to exclude a placebo effect, as blinding could not be performed during the exercise intervention. Future reviews could reduce the heterogeneity between included studies by restricting the inclusion criteria more strictly.

## Conclusion

This meta-analysis revealed that exercise had beneficial effects in improving balance, walking ability, walking endurance, fatigue, and quality of life in people with MS. The effect of exercise on improving fatigue was associated with the age of the participants, with the younger the age, the greater the improvement in fatigue. To improve fatigue and quality of life, this meta-analysis provides clinicians with evidence to recommended that people with MS participate in resistance exercise and aerobic exercise, respectively.

## Data availability statement

The original contributions presented in the study are included in the article/Supplementary material, further inquiries can be directed to the corresponding authors.

## Author contributions

LD: Conceptualization, Data curation, Formal analysis, Investigation, Methodology, Software, Visualization, Writing – original draft, Writing – review & editing. HX: Data curation, Formal analysis, Investigation, Methodology, Software, Writing – review & editing. SZ: Data curation, Formal analysis, Investigation, Methodology, Software, Writing – review & editing. YZ: Data curation, Formal analysis, Investigation, Visualization, Writing – review & editing. XT: Data curation, Formal analysis, Investigation, Writing – review & editing. YL: Data curation, Formal analysis, Investigation, Writing – review & editing. XH: Data curation, Funding acquisition, Investigation, Project administration, Resources, Software, Writing – review & editing. LY: Conceptualization, Funding acquisition, Methodology, Project administration, Resources, Supervision, Validation, Visualization, Writing – review & editing.
