# Supplementary material for: Effects of exercise in people with multiple sclerosis: a systematic review and meta-analysis
Source: Front Public Health. 2024 Apr 10;12:1387658. doi: 10.3389/fpubh.2024.1387658 (PMC11039920; doi:10.3389/fpubh.2024.1387658)
Supplement: Supplementary file 1 [file Data_Sheet_1.docx]

Supplementary Material

# Supplementary Figures and Tables

## Supplementary Figures


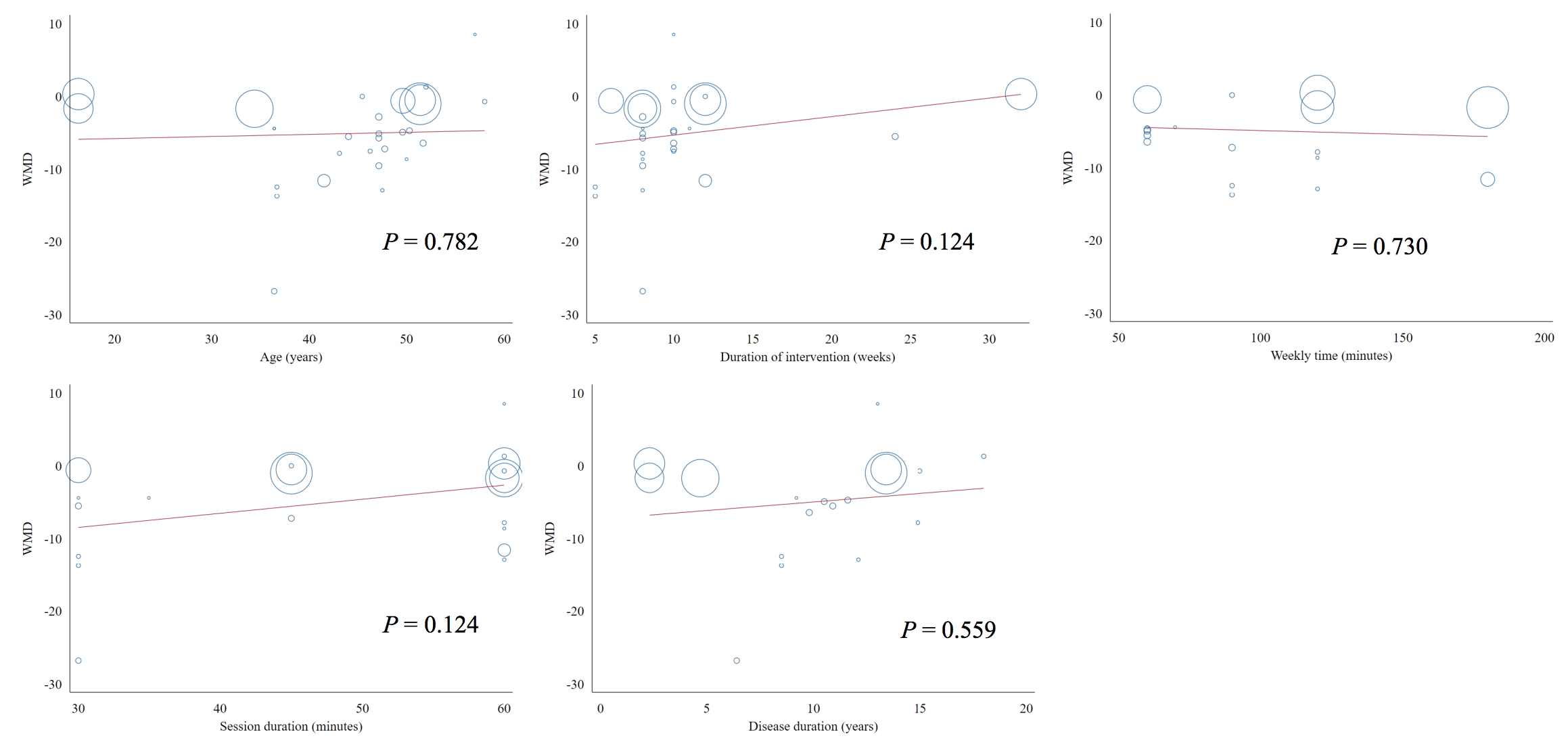


**Supplementary Figure 1.** Meta-regression analyses results of fatigue.


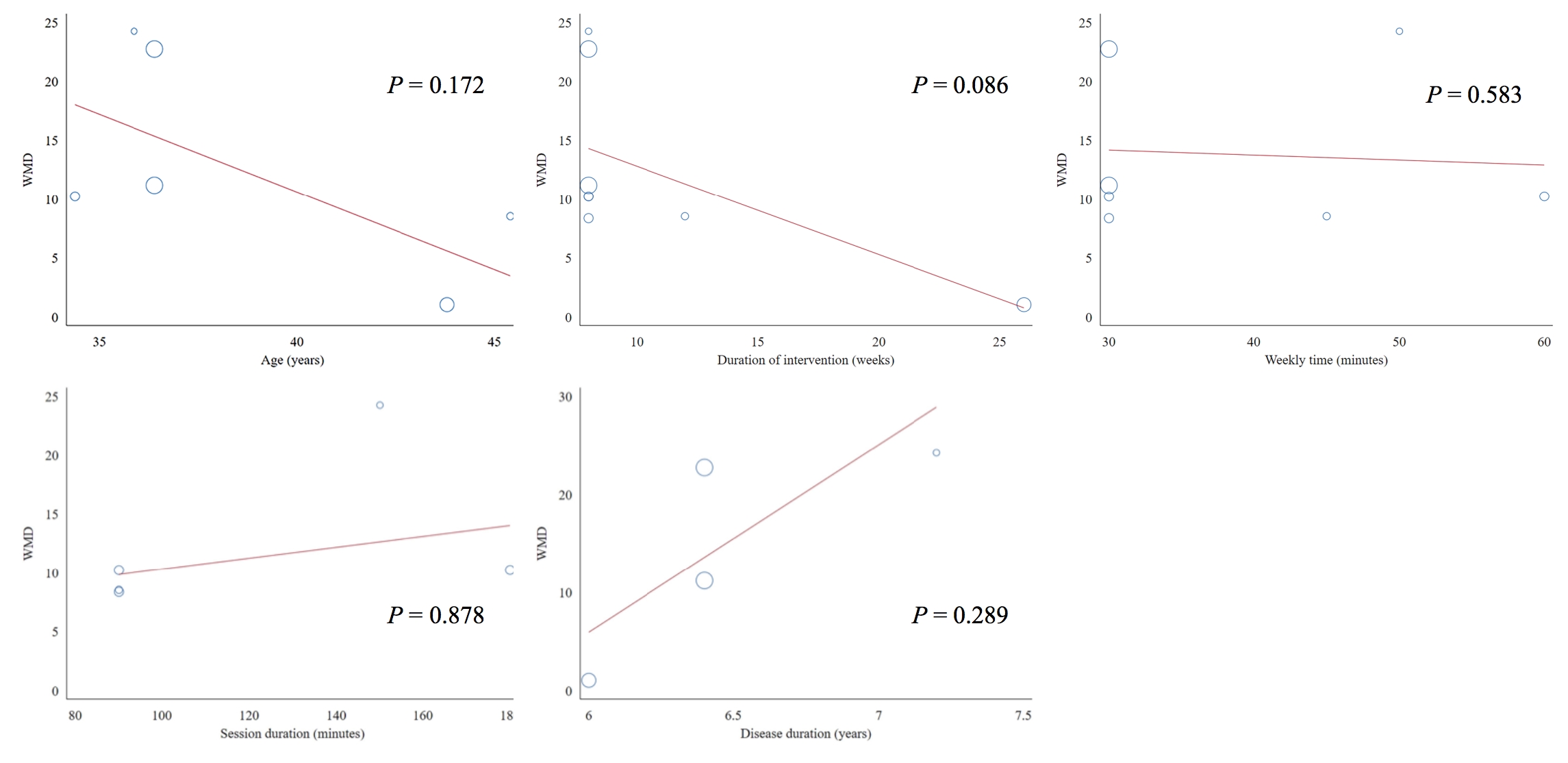


**Supplementary Figure 2.** Meta-regression analyses results of quality of life.


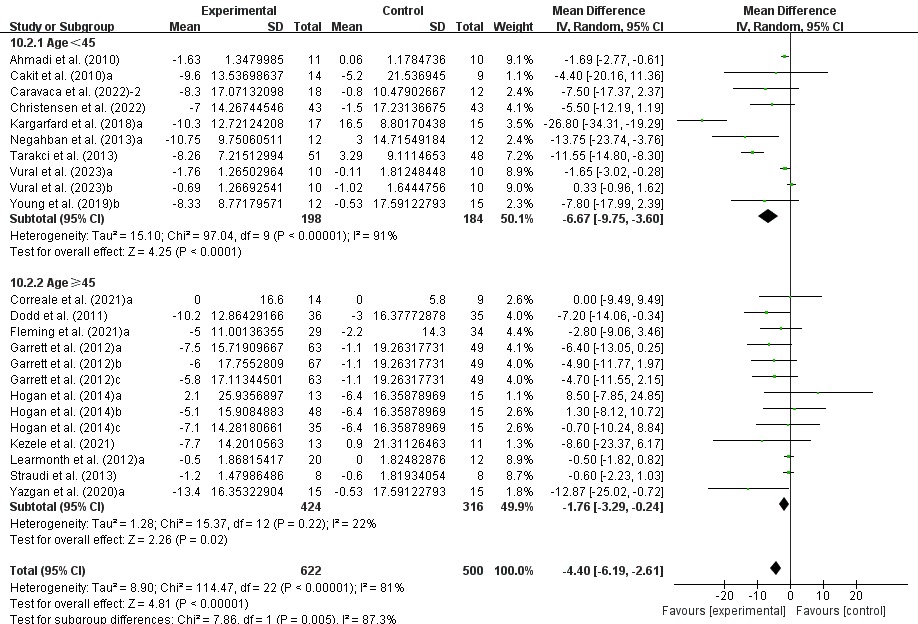


**Supplementary Figure 3.** Meta-analysis of the effect of participants’ age on fatigue in MS patients.


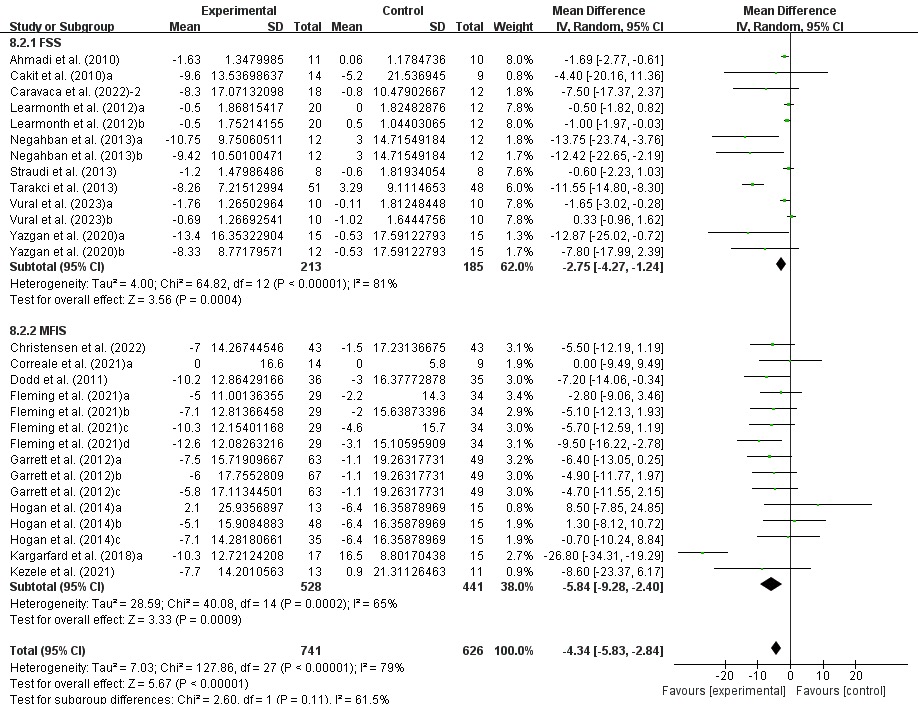


**Supplementary Figure 4.** Meta-analysis of the effect of type of fatigue test on fatigue in MS patients on fatigue in MS patients.


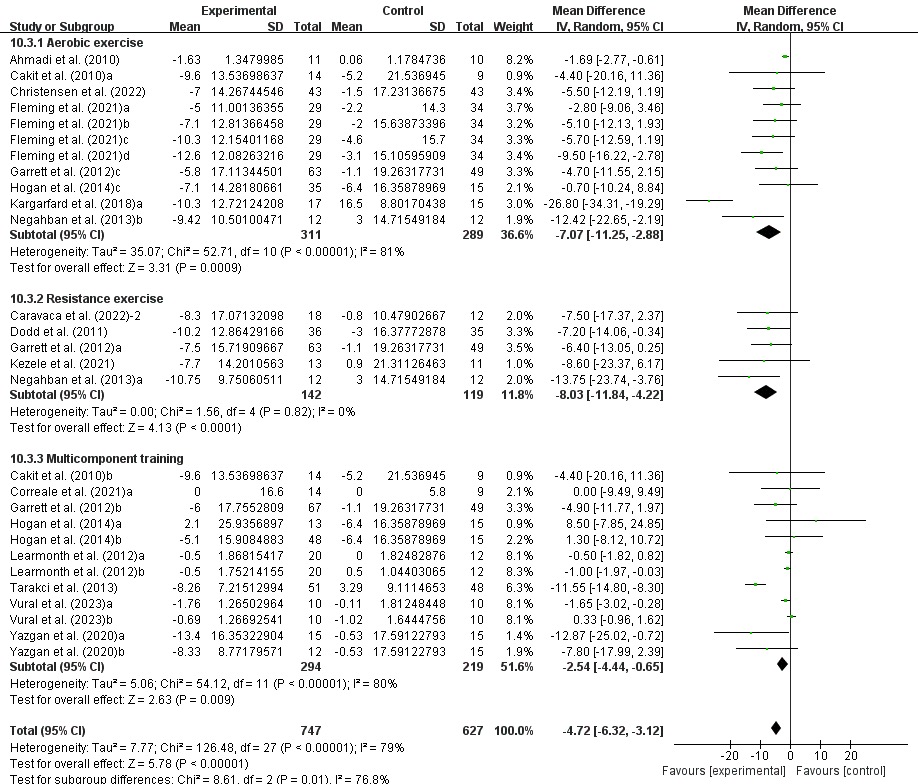


**Supplementary Figure 5.** Meta-analysis of the effect of type of intervention on fatigue in MS patients on fatigue in MS patients.


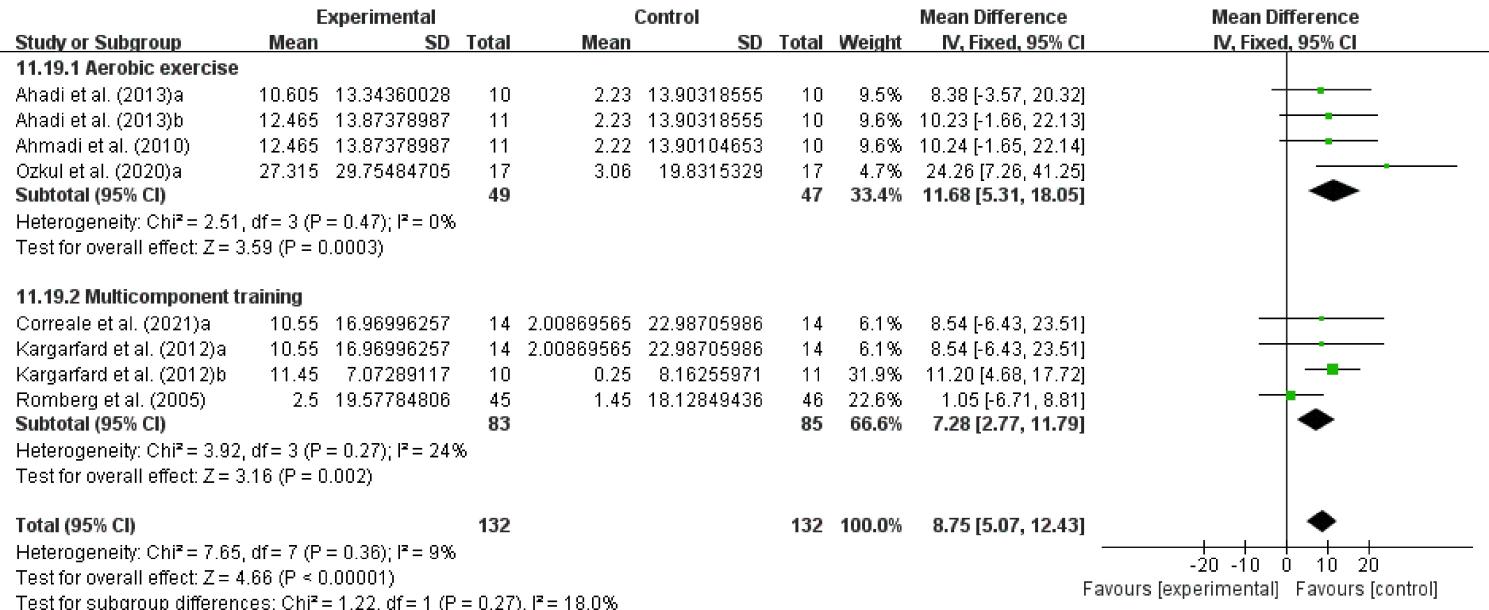


**Supplementary Figure 6.** Meta-analysis of the effect of type of intervention on quality of life in MS patients on fatigue in MS patients.


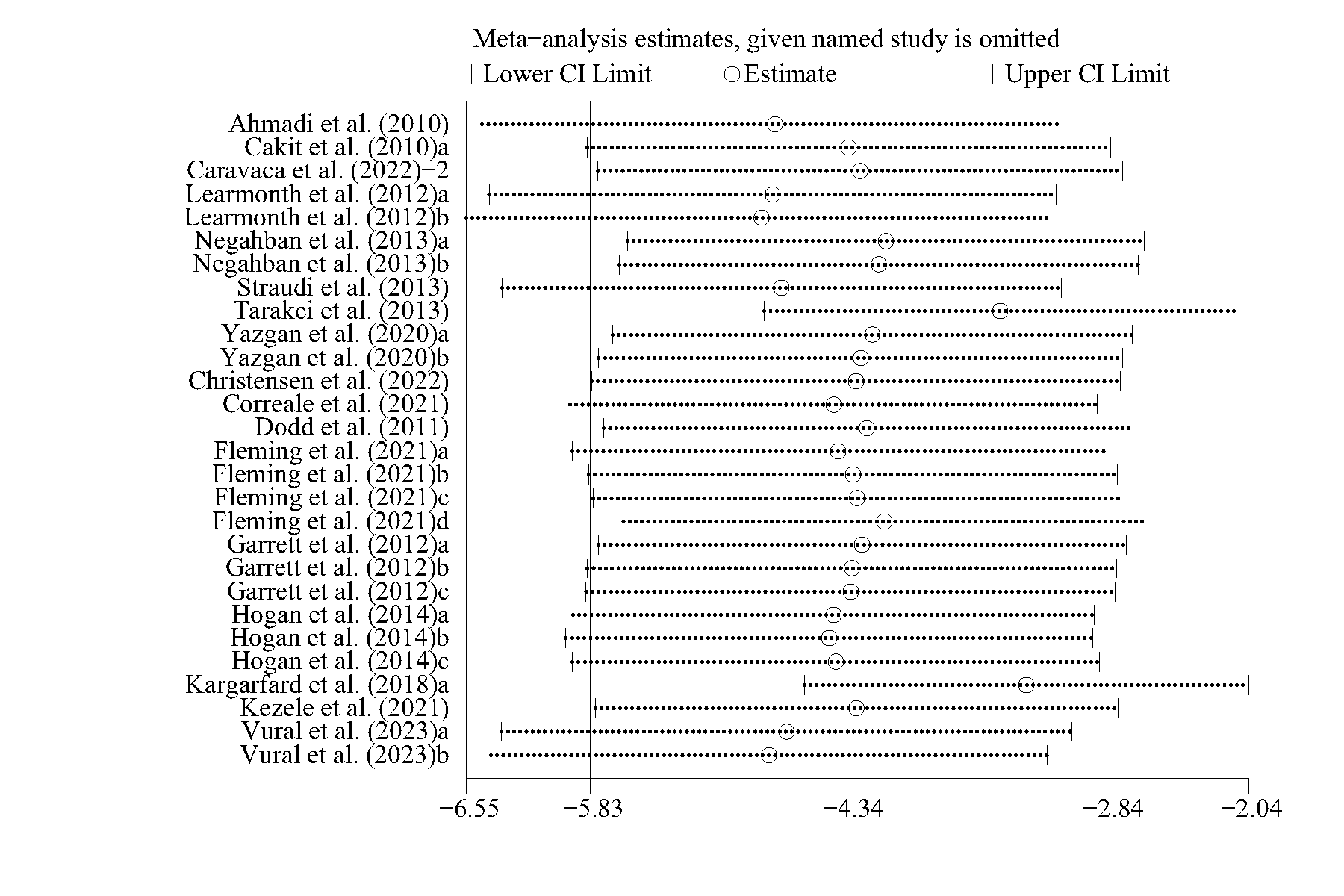


**Supplementary Figure 7.** Sensitivity analyses results of fatigue.


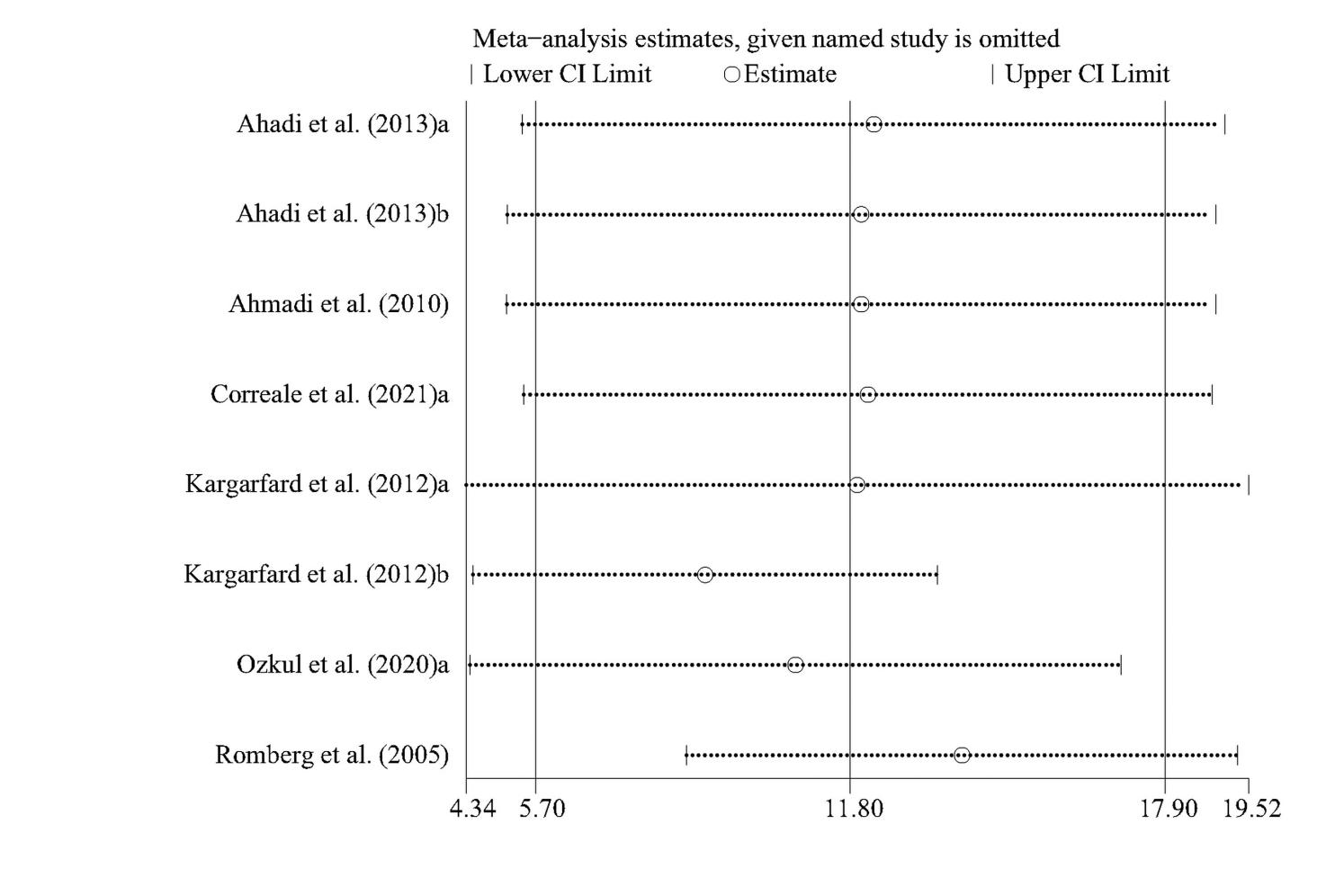


**Supplementary Figure 8.** Sensitivity analyses results of quality of life.


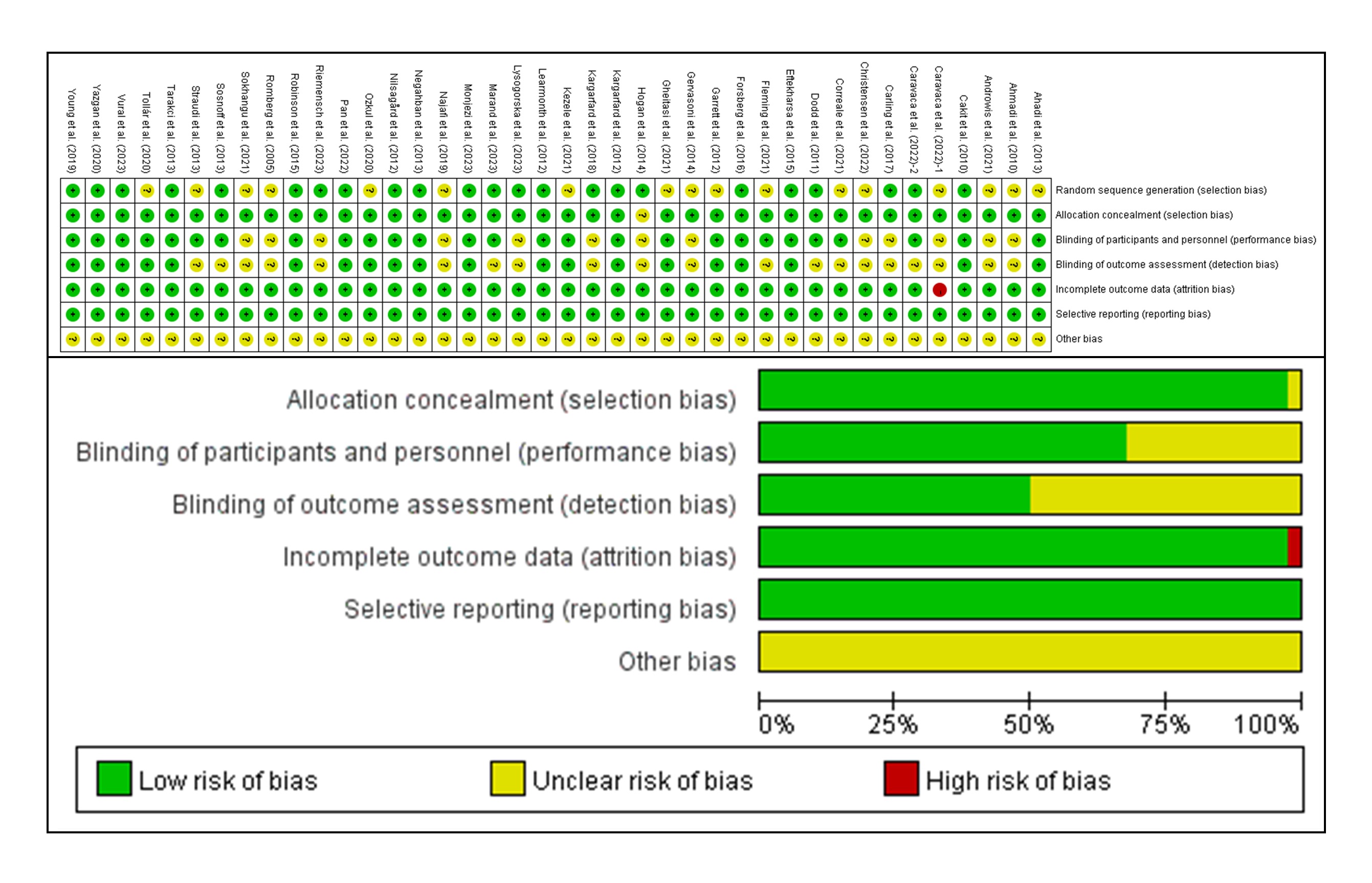


**Supplementary Figure 9.** Results of Cochrane risk of bias tool.


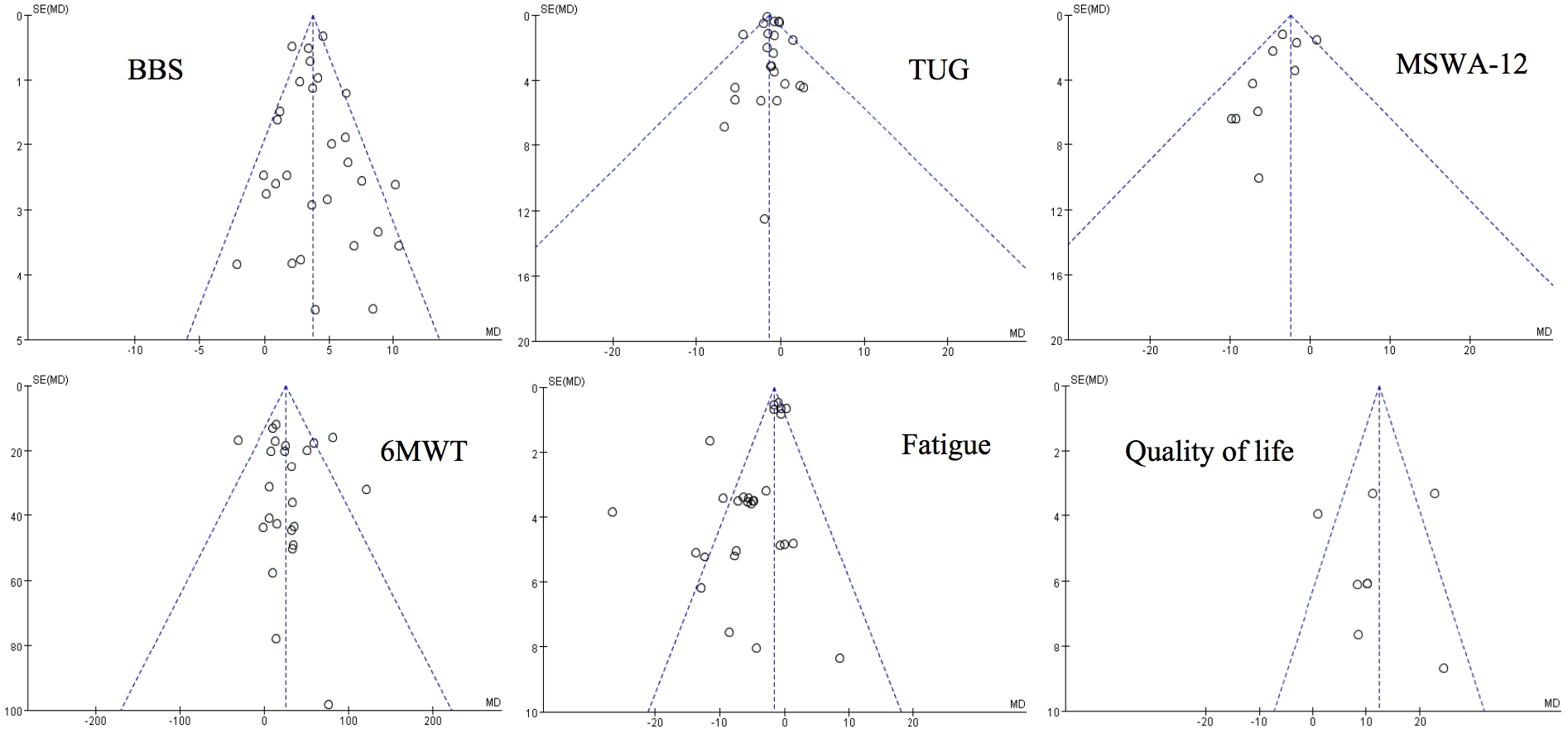


**Supplementary Figure 10.** Funnel plot.

## Supplementary Tables

**Supplementary Table 1.** Search terms for exercise, multiple sclerosis, balance, gait, fatigue, and quality of life.

| **Exercise search terms combined with ‘OR’** |
| --- |
| Physical exercise programs; Physical Therapy Modalities; Physical Therapy Modalities; Modalities, Physical Therapy; Modality, Physical Therapy; Physical Therapy Modality; Physiotherapy; Physical Therapy Techniques; Physical Therapy Technique; Techniques, Physical Therapy; Exercise Movement Techniques; Exercise Movement Techniques; Movement Techniques, Exercise; Exercise Therapy; Exercise Therapy; Therapy, Exercise; Exercise Therapies; Therapies, Exercise; Exercise, Physical; Exercises, Physical; Physical Exercise; Physical Exercises; Exercise, Isometric; Exercises, Isometric; Isometric Exercises; Isometric Exercise; Exercise, Aerobic; Aerobic Exercises; Aerobic Exercise; Resistance Training; Resistance Training; Training, Resistance; Strength Training; Training, Strength; Weight-Lifting Strengthening Program; Strengthening Program, Weight-Lifting; Strengthening Programs, Weight-Lifting; Weight Lifting Strengthening Program; Weight-Lifting Strengthening Programs; Weight-Lifting Exercise Program; Exercise Program, Weight-Lifting; Exercise Programs, Weight-Lifting; Weight Lifting Exercise Program; Weight-Lifting Exercise Programs; Weight-Bearing Strengthening Program; Strengthening Program, Weight-Bearing; Strengthening Programs, Weight-Bearing; Weight Bearing Strengthening Program; Weight-Bearing Strengthening Programs; Weight-Bearing Exercise Program; Exercise Program, Weight-Bearing; Exercise Programs, Weight-Bearing; Weight Bearing Exercise Program; Weight-Bearing Exercise Programs |
| **Multiple sclerosis search terms combined with ‘OR’** |
| Multiple Sclerosis or Sclerosis, Multiple or Sclerosis, Disseminated or Disseminated Sclerosis or MS (Multiple Sclerosis) or Multiple Sclerosis, Acute Fulminating |
| **Balance search terms combined with ‘OR’** |
| Postural Balance or Musculoskeletal Equilibrium or Posture Balance or Equilibrium, Musculoskeletal or Posture Balance or Balance, Posture or Posture Balances or Posture Equilibrium or Equilibrium, Posture or Posture Equilibriums or Postural Equilibrium or Equilibrium, Postural or Balance, Postural or Postural Control or Control, Postural or Postural Controls or Posture Control or Control, Posture or Posture Controls |
| **Gait search terms combined with ‘OR’** |
| Gait or Gaits |
| **Fatigue search terms combined with ‘OR’** |
| Fatigue or Lassitude or tiredness |
| **Quality of life search terms combined with ‘OR’** |
| Quality of Life or Life Quality or Healh-Related Quality Of Life or Health Related Quality Of Life or HRQOL |

**Supplementary Table 2.** Methodological assessment of randomized controlled trials included in the systematic review using the PEDro scale.

| **Study** | **A** | **B** | **C** | **D** | **E** | **F** | **G** | **H** | **I** | **J** | **K** | **Score** |
| --- | --- | --- | --- | --- | --- | --- | --- | --- | --- | --- | --- | --- |
| Ahadi et al. (2013) | Y | 1 | 1 | 1 | 0 | 0 | 0 | 1 | 1 | 1 | 1 | 7/10 |
| Ahmadi et al. (2010) | Y | 1 | 1 | 1 | 0 | 0 | 0 | 1 | 1 | 1 | 1 | 7/10 |
| Androwis et al. (2021) | Y | 1 | 1 | 1 | 0 | 0 | 1 | 0 | 1 | 1 | 1 | 7/10 |
| Cakit et al. (2010) | Y | 1 | 0 | 1 | 1 | 0 | 1 | 0 | 1 | 1 | 1 | 7/10 |
| Caravaca et al. (2022)-1 | Y | 1 | 0 | 1 | 1 | 0 | 1 | 1 | 1 | 1 | 1 | 8/10 |
| Caravaca et al. (2022)-2 | Y | 1 | 0 | 1 | 1 | 0 | 1 | 1 | 1 | 1 | 1 | 8/10 |
| Carling et al. (2017) | Y | 1 | 0 | 1 | 0 | 0 | 0 | 1 | 1 | 1 | 1 | 6/10 |
| Christensen et al. (2022) | Y | 1 | 1 | 1 | 0 | 0 | 0 | 0 | 1 | 1 | 1 | 6/10 |
| Correale et al. (2021) | Y | 1 | 1 | 1 | 0 | 0 | 0 | 1 | 1 | 1 | 1 | 7/10 |
| Dodd et al. (2011) | Y | 1 | 0 | 1 | 0 | 0 | 0 | 1 | 1 | 1 | 1 | 6/10 |
| Kargarfard et al. (2012) | Y | 1 | 1 | 1 | 0 | 0 | 0 | 1 | 1 | 1 | 1 | 7/10 |
| Fleming et al. (2021) | Y | 1 | 1 | 1 | 0 | 0 | 0 | 0 | 1 | 1 | 1 | 6/10 |
| Forsberg et al. (2016) | Y | 1 | 0 | 1 | 1 | 0 | 1 | 0 | 1 | 1 | 1 | 7/10 |
| Garrett et al. (2012) | Y | 1 | 1 | 1 | 0 | 0 | 0 | 0 | 1 | 1 | 1 | 6/10 |
| Gervasoni et al. (2014) | Y | 1 | 1 | 1 | 0 | 0 | 0 | 1 | 1 | 1 | 1 | 7/10 |
| Eftekharsa et al. (2015) | Y | 1 | 0 | 1 | 0 | 0 | 0 | 1 | 1 | 1 | 1 | 7/10 |
| Gheitasi et al. (2021) | Y | 1 | 1 | 1 | 0 | 0 | 0 | 1 | 1 | 1 | 1 | 7/10 |
| Hogan et al. (2014) | Y | 1 | 0 | 1 | 0 | 0 | 0 | 1 | 1 | 1 | 1 | 5/10 |
| Kargarfard et al. (2018) | Y | 1 | 0 | 1 | 0 | 0 | 0 | 0 | 1 | 1 | 1 | 7/10 |
| Kezele et al. (2021) | Y | 1 | 1 | 1 | 0 | 0 | 0 | 1 | 1 | 1 | 1 | 7/10 |
| Learmonth et al. (2012) | Y | 1 | 0 | 1 | 0 | 0 | 0 | 1 | 1 | 1 | 1 | 6/10 |
| Najafi et al. (2019) | Y | 1 | 1 | 1 | 0 | 0 | 0 | 1 | 1 | 1 | 1 | 7/10 |
| Negahban et al. (2013) | Y | 1 | 0 | 1 | 0 | 0 | 0 | 1 | 1 | 1 | 1 | 6/10 |
| Ozkul et al. (2020) | Y | 1 | 0 | 1 | 1 | 0 | 1 | 1 | 1 | 1 | 1 | 8/10 |
| Pan et al. (2022) | Y | 1 | 0 | 1 | 1 | 0 | 1 | 1 | 1 | 1 | 1 | 8/10 |
| Robinson et al. (2015) | Y | 1 | 1 | 1 | 0 | 0 | 0 | 1 | 1 | 1 | 1 | 7/10 |
| Romberg et al. (2005) | Y | 1 | 1 | 1 | 0 | 0 | 0 | 1 | 1 | 1 | 1 | 7/10 |
| Sokhangu et al. (2021) | Y | 1 | 0 | 1 | 0 | 0 | 0 | 1 | 1 | 1 | 1 | 6/10 |
| Sosnoff et al. (2013) | Y | 1 | 1 | 1 | 0 | 0 | 0 | 1 | 1 | 1 | 1 | 7/10 |
| Straudi et al. (2013) | Y | 1 | 1 | 1 | 0 | 0 | 0 | 1 | 1 | 1 | 1 | 7/10 |
| Tarakci et al. (2013) | Y | 1 | 0 | 1 | 0 | 0 | 0 | 1 | 1 | 1 | 1 | 6/10 |
| Tollár et al. (2020) | Y | 1 | 0 | 1 | 1 | 0 | 1 | 1 | 1 | 1 | 1 | 8/10 |
| Yazgan et al. (2020) | Y | 1 | 0 | 1 | 1 | 0 | 1 | 1 | 1 | 1 | 1 | 8/10 |
| Nilsagård et al. (2012) | Y | 1 | 0 | 1 | 0 | 0 | 0 | 1 | 1 | 1 | 1 | 6/10 |
| Young et al. (2019) | Y | 1 | 0 | 1 | 1 | 0 | 1 | 1 | 1 | 1 | 1 | 8/10 |
| Lysogorska et al. (2023) | Y | 1 | 1 | 1 | 0 | 0 | 0 | 1 | 1 | 1 | 1 | 7/10 |
| Marand et al. (2023) | Y | 1 | 0 | 1 | 1 | 0 | 1 | 1 | 1 | 1 | 1 | 8/10 |
| Monjezi et al. (2023) | Y | 1 | 1 | 1 | 0 | 0 | 0 | 1 | 1 | 1 | 1 | 7/10 |
| Riemensch et al. (2023) | Y | 1 | 0 | 1 | 1 | 0 | 1 | 1 | 1 | 1 | 1 | 8/10 |
| Vural et al. (2023) | Y | 1 | 1 | 1 | 0 | 0 | 0 | 1 | 1 | 1 | 1 | 7/10 |

A, eligibility criteria; B, random allocation; C, concealed allocation; D, baseline comparability; E, blind subjects; F, blind therapists; G, blind assessors; H, adequate follow-up; I, intention-to-treat analysis; J, between-group comparisons; K, point estimates and variability. The total score represents the score of the PEDro scale. Item 1 was not scored. Y: yes

**Supplementary Table 3.** Results of egger’s test.

| **Measurements** | **Std_EFF** | **Coef.** | **Std. Err.** | **t** | **P > \|t\|** | **95% CI** |
| --- | --- | --- | --- | --- | --- | --- |
| **TUG** | Slope | -1.387424 | 0.1912732 | -7.25 | 0.000 | -1.785199, -0.9896498 |
|  | Bias | 0.1811921 | 0.3181303 | 0.57 | 0.575 | -0.480396, 0.8427802 |
| **BBS** | Slope | 3.625115 | 0.4146737 | 8.74 | 0.000 | 2.774275, 4.475955 |
|  | Bias | 0.1496077 | 0.3911033 | 0.38 | 0.705 | -0.6528699, 0.9520853 |
| **6MWT** | Slope | 18.60158 | 14.19755 | 1.31 | 0.204 | -10.84233, 48.0455 |
|  | Bias | 0.3403089 | 0.6160306 | 0.55 | 0.586 | -0.9372604, 1.617878 |
| **MSWS-12** | Slope | -0.4827709 | 1.342891 | -0.36 | 0.730 | -3.658204, -2.692662 |
|  | Bias | -0.9801616 | 0.5834149 | -1.68 | 0.137 | -2.359719, 0.3993953 |
| **Fatigue** | Slope | -0.1124413 | 0.6084575 | -0.18 | 0.855 | -1.363144, 1.138261 |
|  | Bias | -1.656259 | 0.473558 | -3.50 | 0.002 | -2.629672, -0.6828469 |
| **Quality of life** | Slope | 14.77087 | 9.412732 | 1.57 | 0.168 | -8.261257, 37.80299 |
|  | Bias | -0.5512121 | 1.985984 | -0.28 | 0.791 | -5.410739, 4.308315 |

Abbreviations: TUG, timed up and go test; BBS, Berg balance scale; 6MWT, 6-minute walk test; MSIS-12, The 12-Item MS Walking Scale; Coef., coefficient; Std. Err., standard error; t, t-test statistic; CI, Confidence Interval.
